# Clinical decision support improves the appropriateness of laboratory test ordering in primary care without increasing diagnostic error: the ELMO cluster randomized trial

**DOI:** 10.1186/s13012-020-01059-y

**Published:** 2020-11-04

**Authors:** Nicolas Delvaux, Veerle Piessens, Tine De Burghgraeve, Pavlos Mamouris, Bert Vaes, Robert Vander Stichele, Hanne Cloetens, Josse Thomas, Dirk Ramaekers, An De Sutter, Bert Aertgeerts

**Affiliations:** 1 Department of Public Health and Primary Care, KU Leuven, Kapucijnenvoer 33 Blok J PB 7001, B-3000 Leuven, Belgium; 2 Department of Public Health and Primary Care, Ghent University, C. Heymanslaan 10, 9000 Ghent, Belgium; 3 Department of Basic and Applied Medical Sciences, Ghent University, C. Heymanslaan 10, 9000 Ghent, Belgium; 4 Center for General Practice, University of Antwerp, Gouverneur Kinsbergen Centrum, Doornstraat 331, 2610 Wilrijk, Belgium; 5 PharmaCS, Merchtem, Belgium

## Abstract

**Background:**

Inappropriate laboratory test ordering poses an important burden for healthcare. Clinical decision support systems (CDSS) have been cited as promising tools to improve laboratory test ordering behavior. The objectives of this study were to evaluate the effects of an intervention that integrated a clinical decision support service into a computerized physician order entry (CPOE) on the appropriateness and volume of laboratory test ordering, and on diagnostic error in primary care.

**Methods:**

This study was a pragmatic, cluster randomized, open-label, controlled clinical trial.

**Setting:**

Two hundred eighty general practitioners (GPs) from 72 primary care practices in Belgium.

**Patients:**

Patients aged ≥ 18 years with a laboratory test order for at least one of 17 indications: cardiovascular disease management, hypertension, check-up, chronic kidney disease (CKD), thyroid disease, type 2 diabetes mellitus, fatigue, anemia, liver disease, gout, suspicion of acute coronary syndrome (ACS), suspicion of lung embolism, rheumatoid arthritis, sexually transmitted infections (STI), acute diarrhea, chronic diarrhea, and follow-up of medication.

**Interventions:**

The CDSS was integrated into a computerized physician order entry (CPOE) in the form of evidence-based order sets that suggested appropriate tests based on the indication provided by the general physician.

**Measurements:**

The primary outcome of the ELMO study was the proportion of appropriate tests over the total number of ordered tests and inappropriately not-requested tests. Secondary outcomes of the ELMO study included diagnostic error, test volume, and cascade activities.

**Results:**

CDSS increased the proportion of appropriate tests by 0.21 (95% CI 0.16–0.26, *p* < 0.0001) for all tests included in the study. GPs in the CDSS arm ordered 7 (7.15 (95% CI 3.37–10.93, *p* = 0.0002)) tests fewer per panel. CDSS did not increase diagnostic error. The absolute difference in proportions was a decrease of 0.66% (95% CI 1.4% decrease–0.05% increase) in possible diagnostic error.

**Conclusions:**

A CDSS in the form of order sets, integrated within the CPOE improved appropriateness and decreased volume of laboratory test ordering without increasing diagnostic error.

**Trial registration:**

ClinicalTrials.gov Identifier: NCT02950142, registered on October 25, 2016

**Supplementary Information:**

The online version contains supplementary material available at 10.1186/s13012-020-01059-y.

Contributions to the literature

- This study shows that a clinical decision support system in the form of order sets for 17 common indications in primary care improved appropriateness and reduced volume of laboratory test ordering.
- It demonstrated that this system was safe as it was non-inferior to usual care on the incidence of diagnostic error.
- It identified some key challenges to using routinely collected data in primary care in implementation science studies such as cluster randomized trials.

## Introduction

Laboratory test ordering is a vital clinical procedure performed in primary care and the number of tests ordered annually is steadily increasing. For 2018, spending on laboratory testing in healthcare has been valued at $ 80 billion in the USA, and since 2013, costs for laboratory spending have increased more than 15%, representing the largest increase in utilization of any outpatient procedure [1, 2]. This rise in costs is largely due to an increase in laboratory test ordering and this trend is not limited to the USA. For instance, in the UK, laboratory test ordering has increased 8.7% annually and 44,847 laboratory tests were ordered per 10,000 person years in 2015 [3]. For laboratory testing, however, more does not equal better. Many tests are ordered inappropriately, meaning that they are overused, misused, or even underused [4, 5]. Inappropriate tests not only pose problems due to the direct costs they present [6], but also because they cause downstream testing [7], might misdirect or delay diagnostics, and may cause harm [8, 9].

Several factors drive inappropriate laboratory testing, such as the increase in availability of new tests, lack of knowledge of indications or tests, perceived expectations from patients, and fear of liability [10]. Uncertainty and fear of diagnostic error with potential malpractice litigation have been shown to be important but poorly understood attitudes influencing inappropriate overuse of diagnostic procedures [11–13]. Strategies to reduce inappropriate laboratory test ordering in primary care include education, feedback and reminders, guidelines, cost displays, and changes to the order forms [14, 15]. The effects of these interventions vary, and currently, the best available evidence supports the use of combined interventions including at least computerized physician order entry (CPOE) systems and reflex testing practices (such as the automatic ordering of additional tests based on the results of a first test) [16]. Innovative health information technology (IT) interventions, such as clinical decision support systems (CDSS), have widely been cited as promising tools to improve laboratory test ordering behavior [11, 15].

CDSS have shown promising results on improving the appropriateness of clinical study ordering [17] and on reducing overutilization of laboratory tests [16, 18]. Most studies on CDSS in primary care have focused on single conditions or single tests [19–21], but studies that evaluated more comprehensive systems appear to have better results [22–24]. Many studies have used test volume as a measure for appropriateness; however, reducing laboratory test volume may not always improve appropriateness. Under-utilization, found to be as high as 45% in the scarce studies evaluating this phenomenon, remains understudied [4]. To date, the true effects of CDSS on the appropriateness of laboratory test ordering and, more importantly, on clinical outcomes remain unclear. Therefore, we designed the Electronic Laboratory Medicine ordering with evidence-based Order sets in primary care (ELMO) study to evaluate the effects of a combined intervention that integrated a CDSS into a CPOE on the appropriateness and volume of laboratory test ordering, and on diagnostic error in primary care [25].

## Methods

Our study was a pragmatic, cluster randomized, open-label, controlled clinical trial. The methods for this study were previously published [25] and the statistical analysis plan (SAP) is available in Supplement 1. General physicians (GPs) were invited to participate in the study through the clinical laboratories with which they collaborated, and all GPs provided a written consent to participate. They were rewarded for enrolling patients and trial-related tasks but they were not rewarded for using the intervention. Patients provided written consent before enrolment.

### Study design and patients

From December 2017 to June 2018, GPs enrolled patients aged ≥ 18 years with a laboratory test order for at least one of 17 indications: cardiovascular disease follow-up or screening, hypertension, check-up, chronic kidney disease (CKD), thyroid disease, type 2 diabetes mellitus, fatigue, anemia, liver disease, gout, suspicion of acute coronary syndrome (ACS), suspicion of lung embolism, rheumatoid arthritis, sexually transmitted infections (STI), acute diarrhea, chronic diarrhea, and follow-up of medication. The combination of tests ordered together for one or more of the above indications at one given time is further referred to as a laboratory panel. The rationale for choosing these specific indications was based on their relevance for primary care and the availability of clinical practice guidelines on diagnostic testing [25, 26]. All tests were analyzed by one of three different ambulatory clinical laboratories.

### Interventions

The CDSS was integrated into a computerized physician order entry (CPOE) in the form of evidence-based order sets that suggested appropriate tests based on the indication provided by the GP. When starting the order entry within the CPOE, GPs first chose a presenting concern or chronic condition. GPs with access to the CDSS then received a list of suggested tests based on the order sets developed for each of the chosen indications. The CDSS included order sets for presenting complaints and for chronic conditions. The order sets were developed to include multiple clinical presentations for specific indications, such as screening, diagnosis, or follow-up. They were based on clinical practice guidelines developed by the Flemish College of Family Physicians [27, 28] and tailored to the different laboratory workflows. The CDSS allowed the GP to change, add, or delete proposed tests prior to confirming the laboratory test order. Control GPs equally recorded the indications for laboratory test ordering in the CPOE but did not receive suggestions from the CDSS. In order to be able to identify tests that were ordered for indications other than the 17 study indications, GPs flagged panels that included additional indications and were prompted to describe these additional indications in a free text field.

### Randomization and procedures

GPs were randomized to a control group who ordered laboratory tests as usual through a CPOE or to an intervention group who had access to the CPOE with integrated CDSS. The intervention was aimed at the GP, and many GPs worked together in a primary care practice (further referred to as practice); hence, we chose to randomize on the level of the practice rather than on the level of the patient. This clustering avoided contamination between GPs and ensured that patients could not be managed by GPs in both intervention and control arms. All practices were allocated prior to patient enrolment using an electronic random number generator in a 1:1 ratio by an independent statistician. We aimed to stratify practices based on their prior experience with a CPOE, but post hoc, we chose to stratify based on the clinical laboratory with which practices were affiliated. Of the three participating laboratories, one had previously implemented a CPOE and two others had only recently started the implementation; hence, experience with a CPOE was associated with the affiliated laboratory.

All practices received a 1-htraining in the use of the CPOE (with or without CDSS) by qualified personnel. Practices were not blinded to the intervention, nor were patients. All involved researchers, including data managers, statisticians, and monitors, were blinded to the allocations until all data were collected, cleaned, and analyzed.

### Outcomes

The primary outcome of the ELMO study was the proportion of appropriate tests over the total number of ordered tests (which included appropriate tests, inappropriate tests, and also tests that were inappropriate because they were not requested). Hence, for the definition of the primary outcome, three numbers were relevant:

- The number of tests ordered appropriately,
- The number of tests ordered inappropriately, and
- The number of inappropriately not-requested tests. This number was only relevant for diabetes mellitus, CKD, rheumatoid arthritis and thyroid disease.

Per patient, aggregated over panels if multiple panels were available, the primary outcome was defined by the ratio (a)/(a + b + c). This is further referred to as the proportion of appropriate tests. Appropriateness was defined restrictively, where a test with no clear indication was considered inappropriate. In addition, recommended tests not ordered for a specific indication (underutilization) were also considered inappropriate. Appropriateness per indication was defined prior to data analysis and was based on the recommendations from the clinical practice guidelines used to develop the intervention. Hence, appropriateness reflected the tests suggested by the CDSS (appropriate and inappropriate under-utilized tests per indication are available in Supplement 1). GP’s tagged panels that included so-called piggyback tests, or tests that were ordered for another indication that one of the 17 study indications. This allowed separate analyses on panels that did not include any piggyback tests.

Secondary outcomes of the ELMO study included diagnostic error, test volume, and cascade activities. For the assessment of diagnostic error, all new diagnoses were extracted from the EHR using a semi-automated clinical report form [29]. All new diagnoses were evaluated for diagnostic error in relation to the indications for which the laboratory tests were ordered. We defined diagnostic error as any potentially delayed diagnosis as described in the protocol [25]. Diagnostic error was assessed independently by two academic clinicians (ND, VP, BV, or GVP) who were blinded to the allocation. Disagreements were resolved by consensus. Laboratory test volume was assessed as the number of tests per laboratory panel.

### Statistical analysis

The planned statistical analyses were described in the published protocol [25] and are available in Supplement 1. All analyses were performed using SAS® Enterprise Guide version 8.2 software. For the primary outcome, a sample of 35 GPs and 7305 tests would have been sufficient to detect a 10% difference in appropriateness (significance level of 5%, corrected for clustering). However, we aimed to recruit 300 GPs and enroll 12,600 patients based on the power calculations for our secondary outcome (80% power to detect a non-inferiority of a 1% difference in incidence of diagnostic error using a significance level of 5% and correcting for clustering). We were able to recruit 288 GPs from 72 practices who included 10,665 patients; hence, the trial was over-powered for the primary outcome, but slightly underpowered for the secondary outcome.

To assess differences between the allocated groups in the proportion appropriate tests, a logistic generalized estimating equation (GEE) model was used, where the marginal proportions were of interest and not the proportions on patient, GP, or practice level. The logistic GEE model included the allocated group and laboratory as factors and practice as the clustering variable. The effect of the intervention was expressed as the difference in proportions with associated 95% confidence intervals. The proportion of appropriate tests in the two allocated groups was also estimated from the GEE model and presented with their 95% confidence intervals.

The proportion of patients with a missed diagnosis was analyzed by means of a logistic GEE model that included the allocation and laboratory as factors and used the practice as the clustering variable. The proportion of patients with a missed diagnosis and associated 95% confidence intervals were estimated from the model. The non-inferiority limit for missed diagnoses was 1%; hence, the intervention was deemed non-inferior if the difference between the allocated groups (intervention–control) was shown to be less than 1%.

We conducted post hoc sensitivity analyses to investigate potential sources of bias. To assess the effect of age difference between both groups, the planned analysis for the primary outcome was also performed on subgroups of patients stratified by age categories. The analysis was also performed on a subset of the total population where practices with extreme age differences were omitted. To assess potential documentation bias, a comparison of several signal tests was made between subgroups in both arms. For instance, the results of mean value for TSH were compared in the subgroup of thyroid disease patients in both arms, allowing us to evaluate whether both subgroups were comparable. We judged that potential documentation bias would have been most probable in the subgroup of patients for which tests were ordered for a general check-up. Differences in patient characteristics may have been influenced by more accurate clinical coding of indications by GPs in the intervention group. Omitting patients with general check-up as an indication leaves only patients with clearly documented indications. We therefore also analyzed appropriateness in the sub-group of patients without tests ordered for general check-up.

## Results

**Fig. 1:**
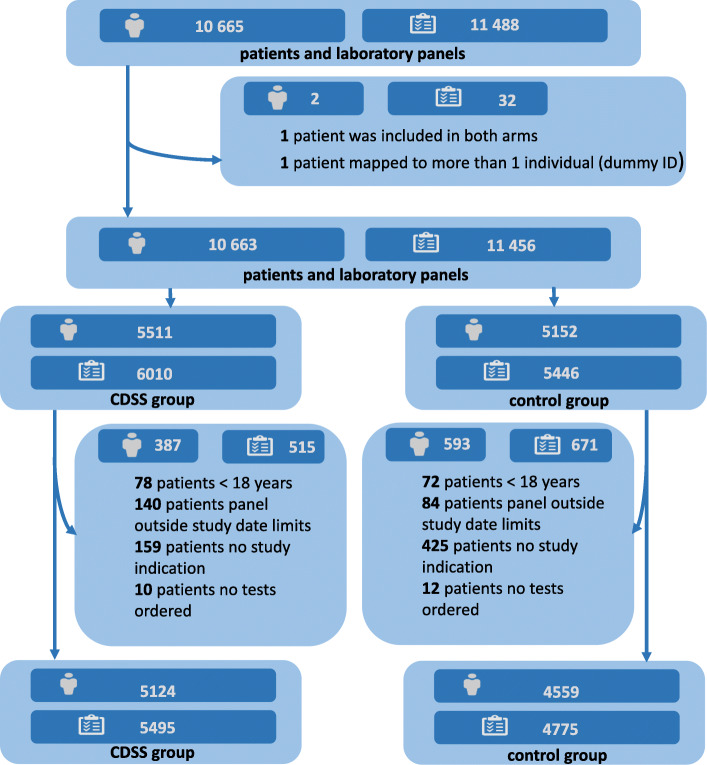
Flow of patient recruitment. CDSS, clinical decision support system; ID, identifier

In total 307 GPs from 76 practices were recruited of which 280 GPs from 72 practices started the study on December 1, 2017. The baseline characteristics of participating GPs are described in eTable 1 of Supplement 2. Eight GPs did not include a single patient or a single laboratory test panel during the trial. eFigure 1 in Supplement 2 shows the flow of GP recruitment prior to the start of the study. Over a period of 7 months, 272 GPs included 10,270 eligible laboratory panels from 9683 patients. Figure 1 illustrates the flow of patients and panels during the study. Baseline patient and GP characteristics are presented in Table 1. Throughout the trial period, 280,804 tests were ordered. No patients or GPs withdrew after the start of the study.

**Table 1:** Demographics of patients. Characteristics of GPs participating in the study and included patients

	CDSS arm	Control arm	Total
Number of GPs	135 (49.63%)	137 (50.37%)	272
Age (mean years, SD)	41 (13.59)	41 (13.27)	
Experience (mean years, SD)	14 (18.81)	15 (19.40)	
% Female	65.00	62.14	
Number of practices per lab	36 (50.00%)	36 (50.00%)	72
Laboratory 1	19 (52.78%)	20 (55.56%)	39
Laboratory 2	5 (13.89%)	3 (8.33%)	8
Laboratory 3	12 (33.33%)	13 (36.31%)	25
Number patients	5124 (52.92%)	4559 (47.08%)	9683
Age (years, SD)	58.33 (17.04)	54.34 (17.61)	56.45 (17.42)
Female sex (*N*, %)	2774 (54.00%)	2578 (56.00%)	5352 (55.10%)
Total number of panels (*N*, %)	5495 (53.51%)	4775 (46.49%)	10,270
Number of panels per indication (*N*, %)^a^			
Check-up	1722 (31.34%)	1936 (40.54%)	3658 (35.62%)
Cardiovascular disease management	1381 (25.13%)	585 (12.25%)	1966 (19.14%)
Hypertension	889 (16.18%)	478 (10.01%)	1367 (13.31%)
Chronic kidney disease	587 (10.68%)	168 (3.52%)	755 (7.35%)
Type 2 diabetes	2160 (39.31%)	953 (19.96%)	3113 (30.31%)
Thyroid disease	1164 (21.18%)	576 (12.06%)	1740 (16.94%)
Sexually transmitted infections	248 (4.51%)	336 (7.04%)	584 (5.69%)
Chronic diarrhea	23 (0.42%)	42 (0.88%)	65 (0.63%)
Acute diarrhea	12 (0.22%)	19 (0.40%)	31 (0.30%)
Acute coronary syndrome	34 (0.62%)	21 (0.44%)	55 (0.54%)
Lung embolism	22 (0.40%)	15 (0.31%)	37 (0.36%)
Rheumatoid arthritis	126 (2.29%)	105 (2.20%)	231 (2.25%)
Medication follow-up	798 (14.52%)	374 (7.83%)	1172 (11.41%)
Gout	170 (3.09%)	39 (0.82%)	209 (2.04%)
Liver disease	416 (7.57%)	157 (3.29%)	573 (5.58%)
Anemia	728 (13.25%)	395 (8.27%)	1123 (10.93%)
Fatigue	606 (11.03%)	520 (10.89%)	1126 (10.96%)
Other	434 (7.90%)	621 (13.01%)	1055 (10.27%)

*GP* general practitioner, *CDSS* clinical decision support system, *SD* standard deviation

^a^The percentages reported for the individual indications use the total number of panels as denominator

Laboratory tests ordered for patients in the CDSS arm were more often appropriate than those ordered for patients in the control arm. There was an absolute difference in the proportion of appropriate tests of 0.21 (95% CI 0.16–0.26, *p* < 0.0001) for all tests included in the study. For panels without piggyback tests, the absolute difference in the proportion of appropriate tests was similar (0.19 (95% CI 0.11–0.28, *p* < 0.0001)). The effects of the CDSS was largest for acute diarrhea, rheumatoid arthritis, chronic diarrhea, CKD, and fatigue. The CDSS had a much smaller effect, or even no effect for STI, lung embolism, ACS, and the follow-up of medication. Results for the difference in proportions for each of the indications included in the CDSS are provided in Table 2. Inappropriate under-utilization accounted for 1.12% of inappropriate tests in the CDSS arm and 0.2% in the control arm.

**Table 2:** Effect of CDSS on proportion of appropriate tests. All values are absolute differences with 95% confidence intervals unless specified otherwise

	Proportion appropriate tests		Difference in proportions	***p*** value
	CDSS arm	Control arm		
Primary outcome (all tests)	0.58 (0.54–0.62)	0.38 (0.34–0.41)	0.21 (0.16–0.26)	< 0.0001
Subgroups per indication				
Check-up	0.26 (0.24–0.28)	0.17 (0.16–0.19)	0.08 (0.06–0.11)	< 0.0001
Medication follow-up	0.78 (0.75–0.82)	0.74 (0.70–0.78)	0.04 (0.00–0.09)	0.0591
Cardiovascular disease management	0.41 (0.37–0.45)	0.30 (0.28–0.32)	0.11 (0.07–0.15)	< 0.0001
Hypertension	0.47 (0.43–0.50)	0.39 (0.35–0.42)	0.08 (0.03–0.12)	0.0007
Type 2 diabetes	0.51 (0.47–0.54)	0.38 (0.35–0.41)	0.13 (0.08–0.17)	< 0.0001
Fatigue	0.81 (0.79–0.83)	0.67 (0.64–0.70)	0.14 (0.10–0.17)	< 0.0001
Anemia	0.82 (0.81–0.84)	0.76 (0.74–0.78)	0.06 (0.03–0.09)	< 0.0001
Liver disease	0.56 (0.53–0.59)	0.43 (0.39–0.46)	0.13 (0.08–0.18)	< 0.0001
Gout	0.27 (0.23–0.31)	0.16 (0.14–0.18)	0.11 (0.06–0.16)	< 0.0001
Chronic kidney disease	0.66 (0.61–0.70)	0.51 (0.46–0.56)	0.14 (0.09–0.20)	< 0.0001
Acute coronary syndrome	0.06 (0.05–0.07)	0.04 (0.02–0.05)	0.02 (0.01–0.04)	0.0081
Lung embolism^a^	0.06 (0.02–0.10)	0.02 (0.01–0.04)	0.03 (− 0.01–0.08)	0.1608
Rheumatoid arthritis	0.79 (0.76–0.82)	0.61 (0.56–0.66)	0.18 (0.12–0.24)	< 0.0001
Thyroid disease	0.50 (0.47–0.54)	0.45 (0.42–0.49)	0.05 (0.01–0.09)	0.0136
Sexually transmitted infections	0.29 (0.23–0.36)	0.33 (0.27–0.39)	− 0.04 (− 0.13–0.06)	0.4719
Acute diarrhea	0.54 (0.46–0.62)	0.33 (0.28–0.38)	0.22 (0.10–0.33)	0.0002
Chronic diarrhea	0.41 (0.34–0.49)	0.25 (0.22–0.29)	0.16 (0.08–0.24)	0.0001

*CDSS* clinical decision support system

^a^Numbers do not include corrections for the laboratory and the number of study indications per panel

CDSS significantly decreased the number of tests per panel. GPs in the CDSS arm ordered 24 (24.02 (95% CI 21.50-26.54)) tests per panel whereas the GPs in the control arm ordered 31 (31.17 (95% CI 28.35–33.99)) tests per panel. This resulted in an absolute decrease of 7 (7.15 (95% CI 3.37–10.93, *p* = 0.0002)) tests per panel.

There was no difference between the CDSS and control group in the proportion of patients with a possible diagnostic error. Eight thousand one hundred sixty-nine new diagnoses were assessed for possible diagnostic error. eFigure 2 in Supplement 2 illustrates the flow of analyzed patients for diagnostic error. In the CDSS arm 2.4% (2.40% (95% CI 2.00–2.80%)) of the patients had a possible diagnostic error and 3% (3.04% (95% CI 2.48–3.61%)) of the patients in the control arm. The absolute difference in proportions was a decrease of 0.66% (95% CI 1.4% decrease–0.05% increase) in possible diagnostic error.

The GPs allocated to the CDSS arm recruited more patients into the study and these patients were on average 4 years older than the patients recruited by the GPs allocated to the control arm. When analyzing the age difference between all patients for which GPs ordered laboratory tests in the year prior and the year after the start of the study, a similar age difference of four years was noted (see Supplement 2), suggesting that the GPs in the CDSS arm treated older patients compared to the GPs in the control arm. In a post hoc sensitivity analysis, stratification by age did not significantly influence the intervention effect on the primary outcome (see eTable 2 in Supplement 2). Omitting practices that were responsible for increasing the average age of patients in the CDSS arm and decreasing the average age of patients in the control arm did not influence the intervention effect either (see eTable 2, eFigures 3-5 in Supplement 2). Including age as a factor in the prespecified primary outcome analysis did not influence the effect estimate. We judged that potential documentation bias would have been highest for the indication “general check-up,” since this was the indication with the largest discrepancy between both arms. Possible documentation bias seemed most probable for patients who control GPs recorded as having no co-morbidities whereas intervention GPs may have been influenced by the CDSS to improve their recording. Leaving out all panels including this indication resulted in a decrease of the intervention effect (difference in proportions of 0.13 (95% CI 0.08–0.17, *p* < 0.0001), but remained significant (see eTable 2 in Supplement 2 for further details). A subgroup analysis of signal tests for the sub-groups general check-up, type 2 diabetes, cardiovascular disease management, thyroid disease, and CKD showed that the patients in both the CDSS as the control arm had comparable values for these signal tests (see eTable 3 in Supplement 2).

## Discussion

To our knowledge, the ELMO study was one of the largest randomized controlled trials to study the effects of a CDSS on laboratory test ordering. The pragmatic design of the study and the novel data collection techniques enabled us to recruit a large number of patients without compromising on data quality [29]. This ELMO study showed that a CDSS for 17 common indications for laboratory test ordering improved appropriateness and reduced volume of laboratory test ordering in primary care without increasing the incidence of diagnostic error. Our CDSS was designed for a wide array of indications and conditions seen in primary care, and the magnitude of the effects witnessed on appropriateness mirrored previous, smaller studies with comprehensive CDSSs [22, 23]. For the indications ACS, STI, and lung embolism, appropriateness was very low and the CDSS had little to no effect on appropriateness. The order sets for these indications were very limited and for ACS and lung embolism, recommended referral to emergency care rather than ordering laboratory tests in primary care. The low rates of appropriateness seem to suggest that when the decision was made to order tests for these indications, GPs ordered many tests associated with risk factors for these conditions rather than only the test(s) to rule in or rule out the condition of the order set. Aside from three indications, we observed that the effect on appropriateness was largest for less frequent indications, such as acute diarrhea, chronic diarrhea, chronic kidney disease, and fatigue. This finding confirms that inappropriateness is more than unnecessary repeat testing but also improper initial testing [4]. Inappropriateness in our study was almost entirely due to over-utilization and the reduction of inappropriateness resulted in an important reduction of the number of ordered tests. Previous studies have used laboratory test volume as a surrogate for appropriateness, and our study confirms that these two outcomes are indeed correlated [14, 22, 30].

Our CDSS was a simple system of order sets, designed to guide GPs in ordering laboratory tests for common indications in primary care. Despite the simplicity of the CDSS, the effects of the intervention were large. We found that GPs in the CDSS arm less frequently ordered tests for general check-up and more frequently for type 2 diabetes and thyroid disease management. One cause for this discrepancy is that the CDSS dissuaded GPs from ordering laboratory tests for general checks. A recent Cochrane systematic review showed that there is no evidence that general checks influence morbidity or mortality and this is mirrored in our CDSS [31]. The limited number of tests included in the order set for general check-up shifted the test ordering behavior of GPs in the CDSS arm. We also found that inappropriate laboratory test ordering was very high compared to similar studies [4, 22]. This is consistent with a recent study on the use of in vitro diagnostics which showed that, compared to other European countries, Belgium has one of the highest rates of diagnostics use per capita [32]. In addition, our restrictive definition to appropriateness will also have influenced this high baseline estimate; however, since the same definition of appropriateness was used in both arms, the absolute difference in proportions between both arms is independent of this estimate.

The CDSS in our study was non-inferior to standard laboratory test ordering. Identifying potential diagnostic error is challenging and variability between clinicians in determining diagnostic error is large [33]. To account for this challenge, we used a multi-stepped approach to determining potential diagnostic error performed by two reviewers independently. We observed low incidences of diagnostic error, consistent with other findings in primary care. Despite being slightly underpowered for this outcome, we found that CDSS did not increase the incidence of diagnostic error. Earlier studies have shown that targeted CDSS for diagnostic testing was effective at reducing diagnostic error [34]. Our study did not aim to show an improvement in diagnostic error, but did aim to show that reducing the volume of testing does not influence diagnostic error.

### Limitations

Our study has several limitations. GPs randomized to the CDSS arm were very similar to those in the control arm; however, patients enrolled in the CDSS arm were on average 4 years older than those enrolled in the control arm. This finding was consistent across all patients managed by the study GPs and was not confined to the study, which suggests that this was not due to selection bias but rather a consequence of the cluster randomization. The older patients in the CDSS arm were more likely to suffer from chronic diseases than patients in the control arm. Since we randomized GPs and not patients, we were unable to use a co-variate constrained randomization approach to minimize these differences in patient baseline characteristics. GPs were not blinded to the intervention and only intervention GPs experienced the effect of selecting an indication on the tests suggested by the CDSS. This may have introduced a certain degree of documentation bias because we assessed the appropriateness of laboratory tests based on the indications reported by the GP during the laboratory test ordering process. We conducted several sensitivity analyses to assess the influence of these possible sources of bias and found that the intervention effect remained robust across these analyses.

We evaluated the effect of our CDSS on the appropriateness of laboratory test ordering; however, the definition of this outcome remains the subject of debate. A comprehensive review of studies on the appropriateness of laboratory test ordering found that many studies lacked valid methods for their definition of appropriateness [5]. Appropriateness in our study was defined as the relevance of the test for the indication or condition for which it was ordered. We used a restrictive definition, which included both overutilization (tests ordered but not indicated) and underutilization (tests indicated but not ordered), but were lenient in considering a test appropriate due to the difficulty of capturing complex clinical scenarios into broad indications. We did not include the timing of repeat testing in our definition which may have resulted in an overestimation of appropriateness for some tests. The assessment of the appropriateness of individual tests for each of the study indications was based on locally available primary care guidelines, which may limit the generalizability of the effects of our CDSS to other settings or even other countries. Furthermore, the trustworthiness of guidelines on diagnostic testing or follow-up of chronic conditions has been shown to be insufficient or even lacking [35]. Nevertheless, we believe that, despite discussions on the appropriateness of individual laboratory tests for specific indications, the relative effects of our CDSS are generalizable to most primary care settings. Another limitation is that we studied the effects of our CDSS for 17 common indications in primary care, and although already very comprehensive, these were not exhaustive. Previous studies have suggested that inappropriateness is influenced by diagnostic uncertainty, suggesting that it may be even more prevalent for rare indications and tests which are not frequently ordered [11].

To determine diagnostic error, our study relied on EHR data. However, previous research has shown that EHR data may not always be reliable for this purpose because formal diagnostic codes may be inconsistent or missing [36]. We had foreseen similar challenges in the data collection for the outcome on the diagnostic error and had planned a chart review in a subset of patients to quantify this problem. Finally, we chose to perform a chart review for all included patients; hence, all diagnoses were a result of a formal chart review rather than an automatic retrieval of diagnostic codes as described in a previous paper [29]. As a result, the only instances of diagnostic error that may have been missed with our methods were situations where the new diagnosis was unknown to the GP and not present in the EHR. This may have influenced the baseline estimate of diagnostic error, which may have been higher than 3% as witnessed in the control arm but should not have influenced the difference between both arms.

### Implications for clinical practice

The results from this study advocate a more wide-scale implementation of our intervention in primary care, certainly on a national level, but also on an international level. Inappropriate laboratory test ordering is not an isolated issue and is common across most high-resource healthcare settings. However, there are certain barriers that may hinder this broader implementation [37, 38]. Our intervention required tailoring to each of the CPOE of the participating laboratories to account for differences in interoperability standards and workflows, which may become a more important barrier when the intervention is implemented across more laboratory information systems. Another important barrier to further implementation and a more sustainable effect is the need for concurrent financial incentives. It is increasingly clear that de-adopting low-value care, such as inappropriate laboratory testing, requires not only evidence-based guidance, but also economic incentives such as value-based payment arrangements [39].

## Conclusions

Our study demonstrated that CDSS improved appropriateness and decreased volume of laboratory test ordering. The magnitude of the effect may have been influenced by high baseline rates of laboratory test ordering and differences in patient characteristics between arms, but the direction of the effect remained robust across sensitivity analyses. We demonstrated that CDSS improved appropriateness of laboratory test ordering for less frequent indications, that are prone to misuse of tests, but also for common indications which are prone to over-utilization. We also demonstrated that CDSS did not increase diagnostic error. Further research is needed to evaluate the effects over longer periods of time, including interventions to improve the sustainability of these effects. In addition, research is needed to evaluate whether systems with a more complex design and more fully integrated in care processes could have a similar effect.

## Supplementary Information

**Additional file 1.**

**Additional file 2.**

## Data Availability

Study data and material are available upon reasonable request from the study authors.
