# Supplementary material for: Clinical decision support improves the appropriateness of laboratory test ordering in primary care without increasing diagnostic error: the ELMO cluster randomized trial
Source: Implement Sci. 2020 Nov 4;15:100. doi: 10.1186/s13012-020-01059-y (PMC7640389; doi:10.1186/s13012-020-01059-y)
Supplement: Supplementary file 1 — Additional file 1. [file 13012_2020_1059_MOESM1_ESM.pdf]

The effect of evidence-based order sets within a CPOE (computerised physician order entry) system on the quantity and quality of laboratory test ordering in family practice: a cluster randomised trial

Data management plan and statistical analysis plan

Chief Investigator:

Prof Dr Bert Aertgeerts
bert.aertgeerts@kuleuven.be
Academic Center for General Practice, KULeuven
Kapucijnenvoer 33, Blok J - box 7001
3000 Leuven

Trial Coordinator:

Nicolas Delvaux
nicolas.delvaux@kuleuven.be
Academic Center for General Practice, KULeuven
Kapucijnenvoer 33, Blok J - box 7001
3000 Leuven

Contents

1. General information ... 3
2. Introduction and objectives ... 7
2.1. Introduction ... 7
2.2. Objectives ... 7
2.2.1. Primary objective: test appropriateness ... 7
2.2.2. Secondary objective: diagnostic error ... 7
2.2.3. Secondary objective: test volume ... 8
2.2.4. Exploratory objective ... 8
2.3. Definitions ... 8
3. Study methods ... 9
3.1. Trial design ... 9
3.2. Randomisation ... 9
3.3. Sample size ... 9
3.4. Timing of analyses ... 9
4. Data capture system ... 10
4.1. Data security ... 10
4.2. Server security ... 11
4.3. Queries ... 11
5. Data monitoring and review ... 11
6. Data management ... 11
6.1. Database structure ... 12
6.1.1. KCELABGP_LAB ... 12
6.1.2. KCELABGP_GP ... 13
6.1.3. KCELABGP_PAT ... 14
6.1.4. KCELABGP_COM ... 14
6.2. Matching of data across different databases ... 14
6.3. Data cleaning ... 15
6.3.1. Inclusion and exclusion criteria ... 15
6.3.2. KCELABGP_LAB ... 15
6.3.3. KCELABGP_GP ... 16
6.4. Data management ... 16
6.4.1. Small cells risk analysis ... 16
6.4.2. Primary outcome appropriateness ... 17
6.4.3. Secondary outcome diagnostic error ... 17
6.4.4. Secondary outcome cascade activities ... 17
7. Database lock ... 17
7.1. Pre-analysis checks ... 17
7.2. Database lock ... 17
7.3. Database unlock ... 17
8. Unblinding ... 18
9. Archiving ... 18

10. Statistical analysis methods 18
10.1. Outcome definitions 18
10.1.1. Primary outcome appropriateness 18
10.1.2. Secondary outcome diagnostic error 33
10.2. Analysis methods 33
10.2.1. Primary outcome analysis 33
10.2.2. Secondary outcome analysis 34
10.2.3. Exploratory outcome analysis 35
10.3. Missing data 35
10.4. Statistical software 35
11. Data archiving 35
12. References 35

1. General information

Full title

The effect of evidence-based order sets within a CPOE (computerised physician order entry) system on the quantity and quality of laboratory test ordering in family practice: a cluster randomised trial

Short title

Evidence-based order sets for laboratory tests

Trial registration

Clinicaltrials.gov: NCT02950142
Sponsor nr: (UZ Leuven and KU Leuven) S59472
KCE: KCE16011

SAP version	v0.1	13/05/2019
	v0.2	19/06/2019
Protocol version	v3.6	07/08/2019
	Amendment	
SAP version history	v0.1	13/05/2019
	v0.2	19/06/2019
	v1.0	18/07/2019

Roles and responsibilities

Chief investigator	Prof Dr Bert Aertgeerts<br>bert.aertgeerts@kuleuven.be<br>**Academic Center for General Practice, KU Leuven**<br>Kapucijnenvoer 33, Blok J - box 7001<br>3000 Leuven
Trial coordinator	Nicolas Delvaux<br>nicolas.delvaux@kuleuven.be<br>**Academic Center for General Practice, KU Leuven**<br>Kapucijnenvoer 33, Blok J - box 7001<br>3000 Leuven
Co-chief investigator	Prof Dr An De Sutter<br>an.desutter@ugent.be<br>**Department of Family Medicine and Primary Health Care, Ghent University**<br>De Pintelaan 185, building K3<br>9000 Ghent
Research assistant	Veerle Piessens<br>veerle.piessens@ugent.be<br>**Department of Family Medicine and Primary Health Care, Ghent University**<br>De Pintelaan 185, building K3<br>9000 Ghent
Research assistant	Tine De Burghgraeve<br>tine.deburghgraeve@kuleuven.be<br>**Academic Center for General Practice, KU Leuven**<br>Kapucijnenvoer 33, Blok J - box 7001<br>3000 Leuven
Statistician	Pavlos Mamouris<br>pavlos.mamouris@kuleuven.be<br>**Academic Center for General Practice, KU Leuven**<br>Kapucijnenvoer 33, Blok J - box 7001<br>3000 Leuven
Chief statistician	Prof Dr Steffen Fieuws<br>steffen.fieuws@kuleuven.be<br>**Leuven Biostatistics and Statistical Bioinformatics Centre, KU Leuven**<br>Kapucijnenvoer 35, Blok D – box 7001<br>3000 Leuven
Data manager	Roel Heylen, Johan Van Bussel<br>roel.heylen@sciensano.be<br>johan.vanbussel@sciensano.be<br>**Healthdata.be (Scientific Institute of Public Health, Sciensano):**<br>Healthdata.be (WIV-ISP)<br>Rue Juliette Wytsmanstraat 14<br>1050 Elsene – Ixelles

Signature page

The undersigned confirm that the following DMP and SAP has been agreed and accepted and that the Chief Investigator agrees to conduct the trial in compliance with the approved protocol and SAP and will adhere to the principles outlined in the requirements for the conduct of clinical trials in the EU as provided for in " Directive 2001/20/EC",), and any subsequent amendments, GCP guidelines, the Belgian law of May 7th 2004 regarding experiments on the human person, the Sponsor's SOPs, and other regulatory requirements as amended.

I agree to ensure that the confidential information contained in this document will not be used for any other purpose other than the evaluation or conduct of the clinical investigation without the prior written consent of the Sponsor

I also confirm that I will make the findings of the study publicly available through publication or other dissemination tools without any unnecessary delay and that an honest accurate and transparent account of the study will be given; and that any discrepancies from the study as planned in this protocol will be explained.

**Trial Coordinator (author):**		
Name: **Nicolas Delvaux**		
Signature:		Date: 18/7/19
**Chief Investigator:**		
Name: **Bert Aertgeerts**		
Signature:		Date: 18/7/19

Abreviations

3BT	Belgian Bilingual Biclassified Thesaurus
CPOE	Computerized Physician Order Entry
CRA	Clinical Research Assistant
DMP	Data Management Plan
ECRF	Electronic Clinical Report Form
EHR	Electronic Health Record
ELMO	Electronic Laboratory Medicine Ordering with evidence-based decision support
GEE	Generalized Estimating Equation
HD	HealthData
HD4DP	HealthData for Data Providers
HD4RES	HealthData for Researchers
ICD	International Classification of Diseases
ICPC	International Classification of Primary Care
ISP-WIV	Institute of Public Health (currently Sciensano)
LIS	Laboratory Information System
LOINC	Logical Observation Identifiers Names and Codes
NICE	National Institute for Health and Care Excellence
PCP	Primary Care Practice
SAP	Statistical Analysis Plan
TTP	Trusted Third Party

2. Introduction and objectives

2.1. Introduction

The Electronic Laboratory Medicine Ordering with evidence-based decision support (ELMO) study aims to investigate the effect of evidence-based decision support (in the form of order sets) on 3 outcomes: volume of laboratory test ordering, appropriateness of laboratory test ordering, and on diagnostic error. An additional exploratory outcome was formulated, being the effect of evidence-based order sets on cascade activities.

This statistical analysis plan (SAP) and data management plan (DMP) specifies and elaborates on the statistical plan described in the full protocol version 3.6 dd 07/08/2019.[1, 2] This report was developed using the reporting standards for SAP.[3]

2.2. Objectives

2.2.1. Primary objective: test appropriateness

To compare the effect of evidence-based order sets versus control on the proportion of appropriate laboratory tests ordered by primary care physicians on 17 common indications for ordering laboratory tests.

Null hypothesis: evidence-based order sets have no effect on the appropriateness of laboratory tests ordered by primary care physicians compared to control.

Alternative hypothesis: evidence-based order sets will increase the proportion of appropriate laboratory tests ordered by primary care physicians from 70% to 80% compared to control.

Population: tests ordered in patients for 17 common indications by primary care physicians

Intervention: Computerized physician order entry (CPOE) with evidence-based order sets

Comparison: CPOE without evidence-based order sets

Outcome: proportion of ordered tests assessed as being appropriate for each of the indications

Time: the effect on the outcome is immediate, at the point of care

2.2.2. Secondary objective: diagnostic error

To demonstrate non-inferiority in the effect of evidence-based order sets versus control on the incidence of missed or delayed diagnoses for 17 common indications by primary care physicians.

Null hypothesis: evidence-based order sets increase the incidence of missed or delayed diagnoses in primary care by more than 1%.

Alternative hypothesis: evidence-based order sets do not worsen the incidence of missed or delayed diagnoses by primary care physicians compared to control, i.e. $pA - pB < 1\%$, with pA and pB being the proportion of missed diagnoses in the intervention and control groups, respectively.

Population: patients for which laboratory tests were ordered for 17 common indications by primary care physicians

Intervention: CPOE with evidence-based order sets

Comparison: CPOE without evidence-based order sets

Outcome: incidence of missed or delayed diagnoses related to the 17 indications

Time: continuously during 6 months following initial laboratory test ordering

2.2.3. Secondary objective: test volume

To compare the effect of evidence-based order sets versus control on the number of laboratory tests ordered by primary care physicians with no restriction on the indications.

Null hypothesis: evidence-based order sets have no effect on the number of laboratory tests ordered by primary care physicians compared to control.

Alternative hypothesis: evidence-based order sets decrease the number of laboratory tests ordered in primary care by 20%.

Population: tests ordered by primary care physicians without restriction on indications

Intervention: CPOE with evidence-based order sets

Comparison: CPOE without evidence-based order sets

2.2.4. Exploratory objective

We will attempt to assess the effect of our intervention on the downstream activities arising from abnormal results of inappropriate tests.

2.3. Definitions

Laboratory panel: the set of ordered laboratory tests ordered by the physician. This panel consists of a series of laboratory tests ordered for one or more indication at one time by a single physician.

Indication: the reason for ordering a series of laboratory tests. A single panel may have more than one indication and sometimes a single test can be ordered for more than one indication.

Study indication: one of the indications included in the study protocol.

Order sets: a set of laboratory tests that are suggested when an indication is selected.

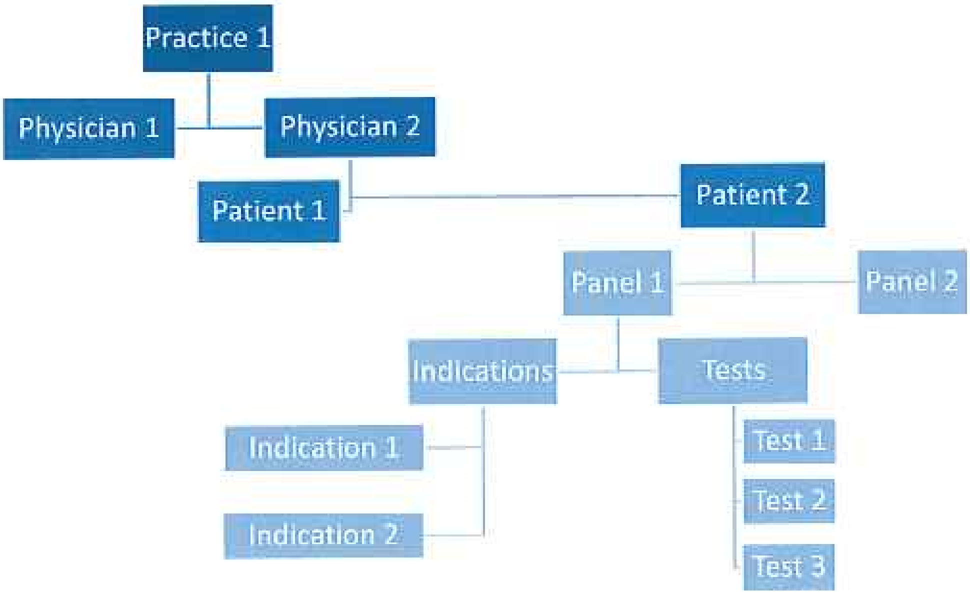

Figure 1: Graphic representation of the relationship between the different clusters and items.

3. Study methods

3.1. Trial design

Our trial is designed as a cluster randomised controlled trial and powered as a superiority trial for our primary outcome. For the secondary outcome, the trial is designed to establish non-inferiority. The trial will have sufficient statistical power for both outcomes and consists of a six-month intervention period and a six-month follow-up period. The trial design with time points can be found in the protocol under 6.

3.2. Randomisation

Participating PCPs were randomised to the intervention or to the control group. The unit of allocation is the PCP. This means that all physicians in the same practice were allocated to the same intervention and that either all or no physicians in the PCP were included in the trial. All patients cared for by the same primary care practice were exposed to the same intervention. We recruited 288 physicians on 01/12/2017 of which 8 physicians dropped out before including patients. The 280 physicians were distributed across 72 PCPs. We stratified the randomisation according to the laboratory with which they collaborated (3 strata). The level of confidence in using a CPOE was associated with the laboratory with which the PCP collaborated. This also assured that all PCPs collaborating with a laboratory were evenly distributed across both arms. Randomisation was done using a random numbers generator by the trial chief statistician. The research team will remain blinded to the allocation until after database lock. Further details on the randomisation scheme can be found in the protocol under 8.3.

3.3. Sample size

For sample size calculations, we refer to the protocol and appendix 3 of the protocol. At the end of the six-month intervention period the planned sample size was verified based on the number of recruited physicians and the average number of patients per physician. With 280 study physicians (clusters), a sample of 12740 patients (45.5 patients on average per physician) would be necessary to have at least 80% power for the secondary outcome (diagnostic error), assuming the original intra-cluster correlation and diagnostic error rates. At that point we had recruited 11200 patients and it was deemed unfeasible to recruit an additional 1500 patients. Since most recruitments were realised by physicians who had already recruited 50 or more patients, the Steering Committee agreed that attempts to recruit additional patients should target physicians with a low number of recruited patients. Therefore, only those physicians who had not yet recruited 10 patients were allowed another month to recruit additional patients. Specific calculations regarding the sample size and the assumed intra-cluster correlations can be found in the protocol under 9.1.

3.4. Timing of analyses

All analyses will be done after database lock. No interim analyses are planned. Database lock is planned in the third quarter of 2019 and analyses are planned subsequently.

Outcome	Origin of data	Timing of data collection
Primary outcome (appropriateness)	Laboratory Information System (LIS)	05-12/2018
Secondary outcome (diagnostic error)	Primary care electronic health record (EHR) and telephone interviews	07/2018-06/2019
Secondary outcome (volume)	LIS	06/2019
Exploratory outcome (cascade activities)	Primary care EHR and LIS	07/2018-06/2019

Table 1: Timing of outcome assessments

4. Data capture system

The data collection tool used in this trial was designed by Healthdata, a division of Sciensano (formerly known as the Scientific Institute for Public Health). The data capture system includes the following:

- Data collection form (HD form)
- Data collection tool (HD4DP)
- Data pseudonymization
- Data encryption
- Data validation tool (HD4RES)
- Datawarehouse

Figure 2 provides a schematic overview of the data capture system and its specific stages.

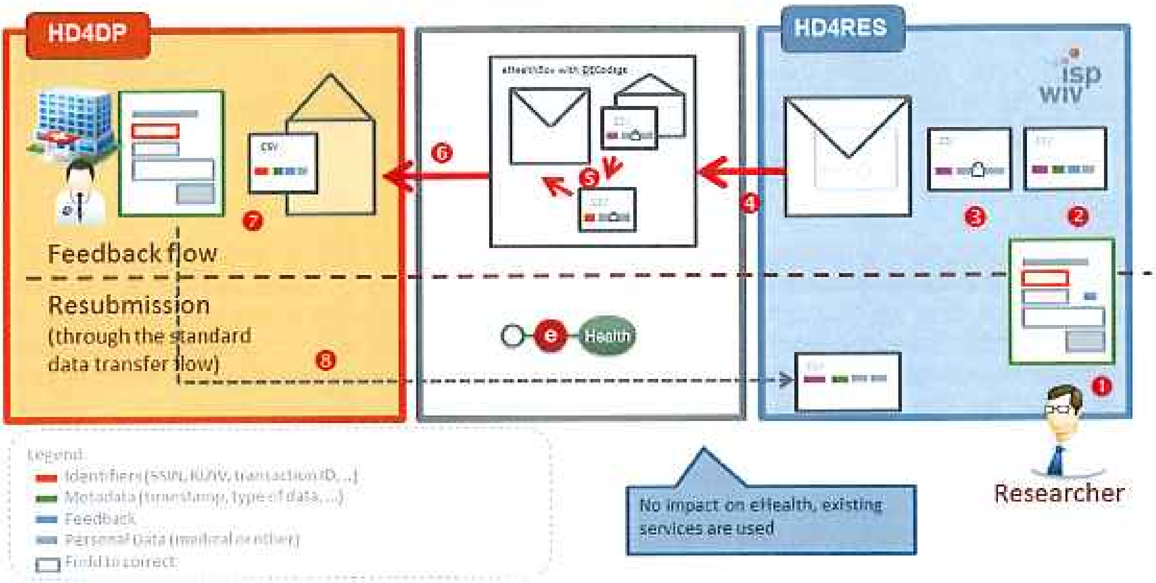

Figure 2: Overview of data capture system. From http://www.Healthdata.be.

The full description of the data capture system has been described in an earlier document[4] and have been reviewed and authorized by the Sector Committee for Social Security and Health of the Privacy Commission on May 16, 2017. In short, the investigators collect study data directly in their primary source. From this primary source a semiautomated export is programmed into the data collection form. For all investigators, the layout of this data collection form is similar. After a first automated data export, specific data elements can be completed manually directly in the data collection form. The form contains optional and mandatory fields and can only be completed when all mandatory fields have been completed. Once completed and validated by the investigator, the form is encrypted and sent to a trusted third party (TTP). For this trial, the eHealth Codage service acts as TTP. The TTP uses a hashing algorithm to pseudonymize the unique identifier of the patient. After this hashing, the data is again encrypted and sent to the Healthdata platform. The data is first received in a validation environment, where it can be viewed, queried and validated. When validated, it can consequently be imported into the data warehouse.

4.1. Data security

Study investigators have access only to the HD4DP tool. This tool is implemented within the EHR or LIS. Access to the tool is granted after the investigator has opened his EHR or has accessed the LIS. No additional authorization is required to access HD4DP. The data collection form is made accessible from within this tool by Healthdata after validation by the research team project manager. At this time, investigators can start collecting data from the primary source and submitting it to Healthdata. Data submitted to Healthdata through HD4DP is encrypted using eHealth encryption tools and can only be decrypted by the eHealth Codage service and Healthdata.

The eHealth Codage service pseudonymizes the patient identifier (social security number) using a hashing algorithm. To prevent security breaches, all patient sensitive data except for the unique identifier

are coded at the primary source. Moreover, except for process logs, no sensitive data is stored at the TTP. After coding of the patient unique identifier, the data is again encrypted using eHealth encryption tools and transferred to Healthdata.

After decryption, the data is stored in the validation environment of Healthdata. Access to the data is managed by Sciensano. The study administrator at Sciensano is responsible for authorization of access after validation by the chief investigator. Access to the data is organized through a two-factor identification including a personal password and an SMS code. Users have differentiated access according to their access/read/write rights depending on their role in the research team.

4.2. Server security

All data are stored on the servers of Healthdata. The servers are hosted at the Arxus Nv datacentre and have been tested and audited by independent engineers. Internal safety measures are under the responsibility of the Healthdata data protection officer. Detailed information on the privacy and safety measures of the Healthdata servers can be found on https://healthdata.sciensano.be/en/information-security-privacy.

4.3. Queries

The data collection form is programmed to allow only structured data and queries are in fact automated on the level of the data collection form. When critical fields are missing or values exceed certain limits, the data collection form will flag data elements and submitting the form will not be possible. At the top of the data collection form for an individual patient, an overview of open comments is visible in a colored line. When all the red fields in the colored line have disappeared, there are no more open comments for the patient, and the form can be submitted.

Once the data for an individual patient is submitted, manual queries can be run in the database to verify data completeness or veracity of the data. A manual query will be programmed to monitor the data collection per physician. Each time the query is run, the results will be documented in a spreadsheet specifically designed to monitor the data collection progression.

5. Data monitoring and review

As specified in the protocol, due to the nature of the collected data (pseudonymised data from data providers), no data monitoring of source documents is possible.

Data collected from the laboratory information systems will be reviewed based on the number of collected informed consent forms. Due to the pseudonymisation of the data in the data collection form, it is not possible to review whether laboratory data for each included patient is collected. We do know how many patients are included per participating physician. This denominator will be used to review the data collection. If for a given physician less data collection forms are collected than the number of informed consent forms in the master file, a notice will be sent to the data provider (laboratory) to review the status of the data collection.

Data collected from the investigators is semi-automatically integrated in the data collection form directly from within the EHR without manual input from the investigator. The protocol specifies that no data monitoring is feasible for this data collection due to the pseudonymized nature of the data. To assist investigators in the use of the data collection form, clinical research assistants (CRAs) are trained in its use in the various EHR systems available in primary care. Differences in database architecture and issues of semantic inoperability across EHR systems could cause the data collection form to misinterpret some of the data entered in the primary source. To avoid this, the CRA's assisted the investigators in the data collection in order to ensure data uniformity and quality. These CRA's have no access to the data within the data warehouse. Again, the denominator of total number in informed consent forms per physician will be used to review the status of the data collection.

For the data collected directly from patients, a CRA is trained to perform structured interviews based on the data elements defined for this data collection. From these structured interviews the data elements will be recorded directly in the data collection form and validated by the CRA. This CRA has no access to the data in the data warehouse.

6. Data management

6.1. Database structure

The database for the ELMO study is maintained by Sciensano (Brussels, Belgium), formerly the Institute of Public Health (ISP-WIV) and structured to be accessible with SAS Enterprise Guide software (Copyright © 2019 SAS Institute Inc.). SAS and all other SAS Institute Inc. product or service names are registered trademarks or trademarks of SAS Institute Inc., Cary, NC, USA.

The database consists of 4 parts:

1. KCELABGP_LAB (data provided by clinical laboratories)
2. KCELABGP_GP (data provided by general practitioners)
3. KCELABGP_PAT (data provided by telephone patient interviews)
4. KCELABGP_COM

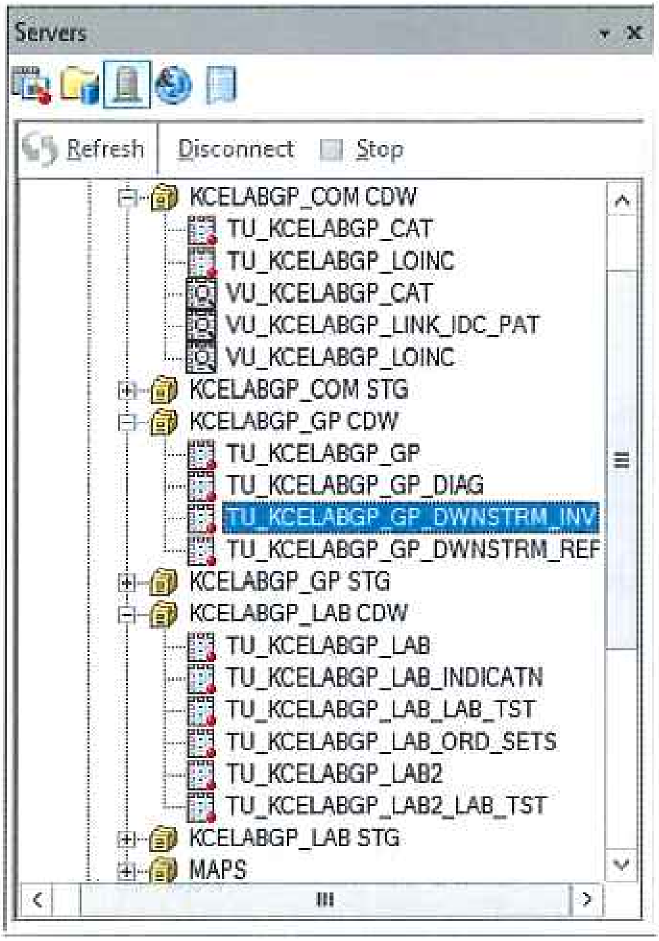

6.1.1. KCELABGP_LAB

The KCELABGP_LAB database contains the data collected through the ambulatory laboratories. This database contains several tables, each containing data from the same data collections. The tables included in the KCELABGP_LAB database are TU_KCELABGP_LAB, TU_KCELABGP_LAB_INDICATN, TU_KCELABGP_LAB_TST, TU_KCELABGP_LAB_ORD_SETS, TU_KCELABGP_LAB2 and TU_KCELABGP_LAB2_LAB_TST.

TU_KCELABGP_LAB

This table contains data on each individual laboratory panel linked to an individual patient. The table includes information on date of birth, sex, reimbursement codes, physician, date of the laboratory test

panel, date of death (if applicable) and total cost of the laboratory test panel. Each row in this table represents a unique laboratory test panel and a single patient may be represented more than once.

TU_KCELABGP_LAB_INDICATN

This table contains data on the indications for each laboratory test panel. It includes data on the selected indications and the additional information provided by the physician when code 99 was used. Code 99 implied that the physicians reported that an indication was applicable outside of the 17 study indications. The exact nature of this indication was included in a free text field of the CPOE. Each row represents a unique indication. Since many laboratory test panels had more than one indication, most panels are represented multiple times.

TU_KCELABGP_LAB_LAB_TST

This table contains data on all ordered laboratory tests. Data on the laboratory test (either a Logical Observation Identifiers Name and Codes (LOINC) code or a Medidoc code), result of the test, upper limit, lower limit are included. Data on whether the result represents a normal or abnormal value is also included. The laboratory test results can be numeric or alphanumeric so that a wide variety of results are possible. Each row in the table represents a unique test. Each panel is represented multiple times as is each patient.

TU_KCELABGP_LAB_ORD_SETS

This table contains data on the selected order sets. For each possible indication, multiple order sets were developed. For instance, for the indication 'hypertension' an order set was developed for the diagnosis of hypertension, for the follow-up of hypertension and so on. This table includes more detailed information on the exact order set that was selected with respect to the indication.

TU_KCELABGP_LAB2 and TU_KCELABGP_LAB2_LAB_TST

This table has an identical structure to the TU_KCELABGP_LAB and TU_KCELABGP_LAB_LAB_TST tables, but contains data on the patients for which cascade tests were ordered.

6.1.2. KCELABGP_GP

The KCELABGP_GP database contains information collected from the electronic health records (EHR) of the physicians on the patients included in the study. This data was collected directly from within the EHR through an electronic clinical report form (eCRF), called an eForm. Each row coincides with an item on this eForm.

TU_KCELABGP_GP

This table contains data on each eForm. Each row corresponds to an individual eForm and contains information on the patient such as sex, date of birth, date of death (if applicable), number of new diagnoses recorded through the eForm, date of the laboratory test panel, information on why the test was ordered and information on any cascade activities.

Each row in this table represents a unique eForm, so for a single patient there may be multiple rows, as for each included laboratory panel a corresponding eForm was collected. Each row has a unique identification key, named 'TXT_WRKFLOW_IDN', through which all data in this database can be linked to an individual eForm.

TU_KCELABGP_GP_DIAG

This table contains data on the new diagnoses recorded through the eForm. Each row represents a unique diagnosis and includes information on the date of the new diagnosis, information on whether the physician considered the diagnosis as related to the laboratory test (either to the indication of the laboratory test order or to the result of one of the laboratory tests in the panel), whether the new diagnosis was diagnosed timely, the name of the new diagnosis and International Classification of Primary Care (ICPC-2) or International Classification of Diseases (ICD-10) codes.

TU_KCELABGP_GP_DWNSTRM_INV

This table contains data on all the downstream investigations recorded through the eForms. Each row represents a unique downstream investigation and includes information on the patient, the type of investigation and free text additions registered by the physician.

TU_KCELABGP_GP_DOWNSTRM_REF

This table contains information on the downstream referrals recorded in the eForm. Each row represents a unique downstream referral and includes information on the patient and on the specialty of the specialist or other healthcare professional to whom was referred.

6.1.3. KCELABGP_PAT

TU_KCELABGP_PAT

This table is identical in structure to that in the KCELABGP_GP database.

TU_KCELABGP_PAT_DIAG

This table is identical in structure to that in the KCELABGP_GP database.

TU_KCELABGP_PAT_DWNSTRM_INV

This table is identical in structure to that in the KCELABGP_GP database.

TU_KCELABGP_DWNSTRM_REF

This table is identical in structure to that in the KCELABGP_GP database.

TU_KCELABGP_PAT_LAB_TST_ORD

This table is identical in structure to that in the KCELABGP_GP database.

6.1.4. KCELABGP_COM

This database contains tables that are required to be able to map or merge data from the KCELABGP_LAB databases with the KCELABGP_GP databases. The identification key for a patient in the KCELABGP_LAB database is not identical to that in the KCELABGP_GP database (for the same patient). KCELABGP_COM contains the mapping table to link the identification numbers for a single patient across the databases.

6.2. Matching of data across different databases

The data from three different providers and databases are linked through a patient identification key. This allows us to identify the same patient across databases. However, for some patients, multiple laboratory panels are included in the KCELAB_LAB database. Besides the patient identification key, we will also use 'date of laboratory test', a data element present in all three datasets, to link an eForm to its corresponding laboratory panel.

The majority of eForms can be linked automatically based on corresponding patient identifiers and 'date of laboratory test'. However, a substantial number of eForms cannot be linked this way, partly because in the eForms the laboratory date had to be entered manually and was therefore susceptible to typing errors.
We will apply the rules below to be able to manually link the eForms to the corresponding laboratory panels:

a) If the patient identification key and laboratory test date are identical in the laboratory panel (row in KCELAB_LAB database) and the eForm (row in KCELAB_GP database), then the rows will be matched.
b) If the patient identification key between the databases are identical and the laboratory panel date in the eForm falls within a period of 14 days before and up to 14 days of the laboratory panel date in the KCELAB_LAB database, then the rows will be matched.
c) If the patient identification key between the databases are identical and the eForm laboratory date differs exactly 1 year with the laboratory panel date, then the rows will be matched.
d) If the patient identification key between the databases are identical, but the eForm laboratory date has a reverse day and month notification (DDMM) compared to a non-linked laboratory panel date, then the rows will be linked.

We applied the same procedure to link the patient interviews to the corresponding laboratory panels.

6.3. Data cleaning

6.3.1. Inclusion and exclusion criteria

Despite stringent inclusion and exclusion criteria, some patients included in the study do not conform to these predefined criteria. All patients included who are younger than 18 years of age at the start of the study will be excluded from the database. Patients included after 01/06/2018 will be excluded. If a patient had been included at a previous date, laboratory panels included after 01/06/2018 will be excluded. The only exception to this criterium are patients included by physicians who had not included 10 patients on 01/06/2018. They were offered the opportunity to include additional patients throughout June 2018. All patients or laboratory panels included after 01/07/2018 will be excluded.

6.3.2. KCELABGP_LAB

The laboratory tests are coded with two possible coding systems: Medidoc[5] or LOINC codes.[6] All Medidoc codes will be mapped to their corresponding LOINC codes so that the entire database was available in one universal code set. The recoding will be performed semi-automatically. Where possible the ReTaM database of laboratory codes will be used as a mapping table.[7] However, many codes will need to be mapped manually. For this manual mapping two sources will be used: the LOINC reference website (https://search.loinc.org) from the Regenstrief Institute and the latest release of Medidoc codes available from Corilus. For a small set of laboratory tests, the meaning of the original Medidoc code is not retrievable and these tests will not be recoded. Observations that are not laboratory test results will be deleted. This includes observations such as weight, height, body mass index, lipemic index, hemolytic index, and insignificant text information.

If reference values (upper limit and lower limit) of the laboratory result value are missing, these will be imputed. If possible, the reference values provided by the laboratory will be used. Sometimes the reference values for the same test differ across laboratories. If reference values are dependent on sex and or age, these variables are considered. Some reference values are dependent on additional clinical information which is not available. For example, the reference value for HbA1c is dependent on whether the test is ordered as a screening test or for the follow-up of a treated diabetes. This

information is not readily available and a reference value that takes both indications into account will be used.

Alphanumeric laboratory results will be recoded to either 'TRUE' (for a normal result), 'FALSE' (for an abnormal result) or 'MISSING' (if the result implies that the test could not be performed). This recoding is done based on the interpretation of the result in relation to the test type. Alphanumeric and numeric results will then be divided into different columns and based on the reference values, the laboratory result will be calculated as 'TRUE' (normal result within the reference value range) or 'FALSE' (abnormal result outside of the reference value range).

Alongside the 17 indications included in the study, physicians had the possibility to add other indications. These indications are included in the database as "99" and a free text field allowed physicians to specify the nature of these indications. In some situations, the free text clearly shows that one or more of the additional indications is in fact one of the study indications. Hence, all references to one or more of the study indications will be recoded from "99" to one of the study indications. Where necessary, a second assessment of the indication will be sought, and if there is any doubt about the indication, it will not be recoded.

6.3.3.KCELABGP_GP

This database contains records that were valid in the past, as well as currently valid records that replace earlier records. Only the currently valid records will be included for further analysis. Furthermore, we will only analyze the data that comes from eForms that can be linked to a corresponding lab panel (see 46.2).

The protocol of this trial states that all new diagnoses made within a period of six months after the laboratory date will be analyzed. Therefore, all diagnoses with a start date before or 6 months after the laboratory date will be excluded. Also diagnoses with unclear labels or where the description clearly indicates that they are not diagnoses but rather treatments or examination will be excluded.

The content of the diagnoses in this table is represented by an ICPC-2 code[8], an ICD-10 code, a free text label or a combination of these three. Since ICPC-2 is the most widely used international classification for systematically recording and analyzing primary care information[i], we will use these codes for further analysis. Therefore, we will complete missing ICPC-2 codes by converting ICD-10 codes or free text labels to their corresponding ICPC-2 code, using the Dutch ICPC-2 and ICPC-ICD10-tables, as well as the Belgian Bilingual Biclassified Thesaurus (3BT), provided by eHealth for all Belgian EHR developers, as conversion tables. All remaining free text labels will be manually recoded to an ICPC-2 code This will result in an additional tailor-made conversion table named 'DIAG_FREETXT'.

6.4. Data management

6.4.1.Small cells risk analysis

A small cells risk analysis (SCRA) was conducted by an independent reviewer and identified three variables that were prone to small cells risk: date of birth of the patient, identification of the physician and postal code of the residence of the patient.

Date of birth of the patient will be coded by changing it to age at the start of the study. As age is an important variable in further statistical analyses, we chose to keep this as a continuous variable rather than an ordinal variable. Further anonymization of this variable would require changing this variable from an age to an age class, hence formatting it from a continuous variable to a class or ordinal variable. This, however, complicates some of the planned data reporting such as basic demographics.

In the original database, each participating physician is identifiable through his or her RIZIV/INAMI number. This variable, combined with age, also compromises the anonymity of the persons in the database. The new physician ID will become an alphanumeric variable that includes a reference to the primary care practice as clustering variable.

Postal code of the place or residence of the patient will be dropped as variable as it is not necessary for further analyses.

6.4.2. Primary outcome appropriateness

For further analyses, some of the tests will be grouped based on the original laboratory test order. Some orders will generate more than one result due to further investigations as a result of an initial abnormal result. For instance, an abnormal result for red blood cells will generate further analyses which will generate more tests. This potentially inflates the number of tests in relation to the number of orders.

6.4.3. Secondary outcome diagnostic error

No further data management is planned for this data.

6.4.4. Secondary outcome cascade activities

No further data management is planned for this data.

7. Database lock

No interim analyses are defined for this study. Only the final analyses defined in the statistical analysis plan (see infra) will be performed.

7.1. Pre-analysis checks

Prior to database lock, based on the data cleaning actions, a final set of patients who meet the inclusion criteria and for whom tests were ordered for one or more of the study indications will be defined. When the last data collection has been completed and when completed data collections forms for at least 85% of the included patients have been imported into the database, a pre-analysis report will be drafted. This document will report on the status of all databases, including:

- Overview of missing forms per physician
- Overview of missing forms per laboratory
- Overview of missing forms per EHR system

If certain outstanding forms are deemed critical for database lock, the investigators responsible for these forms will be contacted to resolve these issues. The outstanding issues and the timeline for these issues will be reported on the pre-analysis report and signed off by the Chief Investigator, Trial Coordinator and Chief Statistician.

Upon resolution of outstanding actions, the database can be locked.

7.2. Database lock

Database lock is foreseen 1 month after the last data collection. The last data collection includes data from the laboratories on cascade laboratory tests. This should allow sufficient time for the data managers to review these data and resolve potential issues. As the database includes several different data collections, we will lock the KCELABGP_LAB database prior to the final data collection on cascade laboratory tests.

Database lock implies a migration of the databases from the validation environment to the analysis environment of the Healthdata data warehouse. In the process of this migration, the recommendations made by the small cells risk analysis will be considered.

Database lock will only take place after all pre-analysis checks have been done and the study team, the Chief Investigator have granted permission. The signed database lock form will confirm that the necessary checks and permissions are in place.

7.3. Database unlock

Database unlock is to be prevented, but in case of critical errors in the database detected after database lock by the statistician. Database unlock must be substantiated in the database unlock form and the required actions be specified. After corrective actions have been performed a new database lock form must be signed before the database lock can be locked again.

8. Unblinding

When the database has been locked, the study team will be unblinded as to the randomization of the PCPs. The Chief Statistician will provide the randomization scheme at which time the study data can be further analyzed. The researchers involved in assessing diagnostic error (see 10.1.2), will not have access to the locked and unblinded database, hence remain blinded to the allocation until all assessments have been made and validated.

9. Archiving

All study data will be archived for a period of 25 years on the servers of Healthdata. Any other study documents, on paper or digital, will be archived at the sponsor site.

Study data at the investigator sites will be stored within the EHR and archived for 25 years according to local primary care procedures.

Informed consent forms and other study documents collected by the laboratories will be archived for a period of 25 years according to local procedures.

10. Statistical analysis methods

10.1. Outcome definitions

10.1.1. Primary outcome appropriateness

We chose to use a restrictive definition to appropriateness as defined by Zhi et al.[9] Appropriateness is therefore directly related to the suggested tests in the study intervention. Tests not included in the study order sets are considered inappropriate. The order sets vary according to specific situations. For example, in some cases, an order set for initial diagnosis was used, but in other situations an order set for follow-up was used. In the composite outcome appropriateness, all tests included in the order sets will be considered appropriate for a given indication. Below, we specify exactly which tests will be considered appropriate for each study indication. Tests in red are considered inappropriate when they are absent from the panel for the given indication. Tests for underutilization were identified through the National Institute for Health and Care Excellence (NICE) quality standards.[10] Those tests considered an essential part of any type of laboratory investigation (diagnosis, follow-up, etc.) for one of the study indications were considered inappropriate when not ordered. We identified tests for inappropriate under-utilization for the indications diabetes mellitus, chronic kidney disease, rheumatoid arthritis and thyroid disease.

Study indication	Test	Code (LOINC)
Cardiovascular risk assessment	Cholesterol total	2093-3
Cardiovascular risk assessment	Cholesterol HDL	2085-9
Cardiovascular risk assessment	Cholesterol total/HDL ratio	32309-7
Cardiovascular risk assessment	Cholesterol total/HDL ratio	9830-1
Cardiovascular risk assessment	Triglycerides	2571-8
Cardiovascular risk assessment	Cholesterol non-HDL	43396-1
Cardiovascular risk assessment	Cholesterol LDL calc	13457-7
Cardiovascular risk assessment	Glucose post meal	16915-1
Cardiovascular risk assessment	Glucose 2h post meal	6689-4
Cardiovascular risk assessment	Glucose fasting	1558-6
Cardiovascular risk assessment	Glucose serum	2345-7
Cardiovascular risk assessment	GGT	2324-2
Cardiovascular risk assessment	ALT (GPT)	1742-6

Cardiovascular risk assessment	CK	2157-6

Table 2: Appropriate tests for the indication 'cardiovascular risk assessment'.

Study indication	Test	Code (LOINC)
Hypertension	Cholesterol total	2093-3
Hypertension	Cholesterol HDL	2085-9
Hypertension	Cholesterol total/HDL ratio	32309-7
Hypertension	Cholesterol total/HDL ratio	9830-1
Hypertension	Triglycerides	2571-8
Hypertension	Cholesterol non-HDL	43396-1
Hypertension	Cholesterol LDL calc	13457-7
Hypertension	Glucose post meal	16915-1
Hypertension	Glucose 2h post meal	6689-4
Hypertension	Glucose fasting	1558-6
Hypertension	Glucose serum	2345-7
Hypertension	GFR MDRD	33914-3
Hypertension	GFR CKD-EPI	62238-1
Hypertension	Creatinine	2160-0
Hypertension	Protein (urine, qualitative)	50949-7
Hypertension	Blood (urine, qualitative)	5794-3
Hypertension	TSH	3016-3
Hypertension	TSH	11580-8
Hypertension	Potassium (venous)	39789-3
Hypertension	Potassium	2823-3
Hypertension	Sodium	2951-2
Hypertension	Metanephrine	21019-5
Hypertension	Normetanephrine	21422-1
Hypertension	Protein/Creatinine ratio (urine)	16285-9

Table 3: Appropriate tests for the indication 'hypertension'.

Study indication	Test	Code (LOINC)
Diabetes mellitus	Cholesterol total	2093-3
Diabetes mellitus	Cholesterol HDL	2085-9
Diabetes mellitus	Cholesterol total/HDL ratio	32309-7
Diabetes mellitus	Cholesterol total/HDL ratio	9830-1
Diabetes mellitus	Triglycerides	2571-8
Diabetes mellitus	Cholesterol non-HDL	43396-1
Diabetes mellitus	Cholesterol LDL calc	13457-7

Diabetes mellitus	Glucose post meal	16915-1
Diabetes mellitus	Glucose 2h post meal	6689-4
Diabetes mellitus	Glucose fasting	1558-6
Diabetes mellitus	Glucose serum	2345-7
Diabetes mellitus	HbA1C (HPLC)	17856-6
Diabetes mellitus	HbA1C (IFCC)	59261-8
Diabetes mellitus	HbA1c	4548-4
Diabetes mellitus	GFR MDRD	33914-3
Diabetes mellitus	GFR CKD-EPI	62238-1
Diabetes mellitus	Creatinine	2160-0
Diabetes mellitus	Potassium	2823-3
Diabetes mellitus	Microalbumin (urine)	14957-5
Diabetes mellitus	Albumine/Creatinine Ratio (urine)	14959-1

Table 4: Appropriate tests for the indication 'diabetes mellitus'.

Study indication	Test	Code (LOINC)
Fatigue	Glucose post meal	16915-1
Fatigue	Glucose 2h post meal	6689-4
Fatigue	Glucose fasting	1558-6
Fatigue	Glucose serum	2345-7
Fatigue	ALT (GPT)	1742-6
Fatigue	GFR MDRD	33914-3
Fatigue	GFR CKD-EPI	62238-1
Fatigue	Creatinine	2160-0
Fatigue	TSH	3016-3
Fatigue	TSH	11580-8
Fatigue	Potassium (venous)	39789-3
Fatigue	Potassium	2823-3
Fatigue	Sodium	2951-2
Fatigue	ESR	4537-7
Fatigue	RBC	789-8
Fatigue	MCH	785-6
Fatigue	MCHC	786-4
Fatigue	MCV	787-2
Fatigue	Hct	789-8
Fatigue	Hct	11273-0
Fatigue	Hct automated	4544-3
Fatigue	Hb	718-7

Fatigue	Hb	785-6
Fatigue	RBC distribution	788-0
Fatigue	WBC	6690-2
Fatigue	WBC formula	49024-3
Fatigue	WBC morphology	18314-5
Fatigue	WBC leucocytes #	6690-2
Fatigue	WBC monocytes %	5905-5
Fatigue	WBC monocytes/leuco	26485-3
Fatigue	WBC monocytes #	26484-6
Fatigue	WBC monocytes # automatc	742-7
Fatigue	WBC lymphocytes %	26478-8
Fatigue	WBC lymphocytes/leuco	26578-8
Fatigue	WBC lymphocytes/leuco autmatic	736-9
Fatigue	WBC lymphocytes #	26474-7
Fatigue	WBC lymphocytes # autmatc	731-0
Fatigue	WBC basophiles #	704-7
Fatigue	WBC basophiles %	26444-0
Fatigue	WBC basophiles/leuco	30180-4
Fatigue	WBC basophiles/100 leuco	706-2
Fatigue	WBC eosinophiles #	711-2
Fatigue	WBC eosinophiles %	26449-9
Fatigue	WBC eosinophiles/leuco	26450-7
Fatigue	WBC eosinophiles/100 leuco	713-8
Fatigue	WBC neutrophiles #	751-8
Fatigue	WBC segm neutrophiles #	30451-9
Fatigue	WBC segm neutrophiles/leuco	26505-8
Fatigue	WBC segm neutrophiles/leuco autmatic	32200-8
Fatigue	WBC normoblasts/leuco	33990-3
Fatigue	WBC normoblasts #	715-3
Fatigue	WBC meylocytes #	30446-9
Fatigue	WBC promyelocytes	26523-1
Fatigue	WBC promyelocytes/leuco	26524-9
Fatigue	WBC metamyelocyten #	30433-7
Fatigue	WBC metamyelocyten/leuco	28541-1
Fatigue	Thrombocytes	777-3
Fatigue	Leuco + Thrombo morphology	48705-8
Fatigue	FT4	14920-3
Fatigue	Ferritin	2276-4

Fatigue	CRP	1988-5
Fatigue	AST (GOT)	1920-8

Table 5: Appropriate tests for the indication 'fatigue'.

Study indication	Test	Code (LOINC)
General check up	Cholesterol total	2093-3
General check up	Cholesterol HDL	2085-9
General check up	Cholesterol total/HDL ratio	32309-7
General check up	Cholesterol total/HDL ratio	9830-1
General check up	Triglycerides	2571-8
General check up	Cholesterol non-HDL	43396-1
General check up	Cholesterol LDL calc	13457-7
General check up	Glucose post meal	16915-1
General check up	Glucose 2h post meal	6689-4
General check up	Glucose fasting	1558-6
General check up	Glucose serum	2345-7

Table 6: Appropriate tests for the indication 'general check up'.

Study indication	Test	Code (LOINC)
Follow-up of medication	Cholesterol total	2093-3
Follow-up of medication	Cholesterol HDL	2085-9
Follow-up of medication	Cholesterol total/HDL ratio	32309-7
Follow-up of medication	Cholesterol total/HDL ratio	9830-1
Follow-up of medication	Triglycerides	2571-8
Follow-up of medication	Cholesterol non-HDL	43396-1
Follow-up of medication	Cholesterol LDL calc	13457-7
Follow-up of medication	GGT	2324-2
Follow-up of medication	ALT (GPT)	1742-6
Follow-up of medication	CK	2157-6
Follow-up of medication	GFR MDRD	33914-3
Follow-up of medication	GFR CKD-EPI	62238-1
Follow-up of medication	Creatinine	2160-0
Follow-up of medication	Potassium (venous)	39789-3
Follow-up of medication	Potassium	2823-3
Follow-up of medication	RBC	789-8
Follow-up of medication	MCH	785-6
Follow-up of medication	MCHC	786-4
Follow-up of medication	MCV	787-2

Follow-up of medication	Hct	789-8
Follow-up of medication	Hct	11273-0
Follow-up of medication	Hct automated	4544-3
Follow-up of medication	Hb	718-7
Follow-up of medication	Hb	785-6
Follow-up of medication	RBC distribution	788-0
Follow-up of medication	WBC	6690-2
Follow-up of medication	WBC formula	49024-3
Follow-up of medication	WBC morphology	18314-5
Follow-up of medication	WBC leucocytes #	6690-2
Follow-up of medication	WBC monocytes %	5905-5
Follow-up of medication	WBC monocytes/leuco	26485-3
Follow-up of medication	WBC monocytes #	26484-6
Follow-up of medication	WBC monocytes # automatc	742-7
Follow-up of medication	WBC lymphocytes %	26478-8
Follow-up of medication	WBC lymphocytes/leuco	26578-8
Follow-up of medication	WBC lymphocytes/leuco autmatic	736-9
Follow-up of medication	WBC lymphocytes #	26474-7
Follow-up of medication	WBC lymphocytes # autmatc	731-0
Follow-up of medication	WBC basophiles #	704-7
Follow-up of medication	WBC basophiles %	26444-0
Follow-up of medication	WBC basophiles/leuco	30180-4
Follow-up of medication	WBC basophiles/100 leuco	706-2
Follow-up of medication	WBC eosinophiles #	711-2
Follow-up of medication	WBC eosinophiles %	26449-9
Follow-up of medication	WBC eosinophiles/leuco	26450-7
Follow-up of medication	WBC eosinophiles/100 leuco	713-8
Follow-up of medication	WBC neutrophiles #	751-8
Follow-up of medication	WBC segm neutrophiles #	30451-9
Follow-up of medication	WBC segm neutrophiles/leuco	26505-8
Follow-up of medication	WBC segm neutrophiles/leuco autmatic	32200-8
Follow-up of medication	WBC normoblasts/leuco	33990-3
Follow-up of medication	WBC normoblasts #	715-3
Follow-up of medication	WBC meylocytes #	30446-9
Follow-up of medication	WBC promyelocytes	26523-1
Follow-up of medication	WBC promyelocytes/leuco	26524-9
Follow-up of medication	WBC metamyelocyten #	30433-7
Follow-up of medication	WBC metamyelocyten/leuco	28541-1

Follow-up of medication	Thrombocytes	777-3
Follow-up of medication	Leuco + Thrombo morphology	48705-8
Follow-up of medication	CRP	1988-5
Follow-up of medication	hCG	19080-1
Follow-up of medication	B-hCG	45194-8

Table 7: Appropriate tests for the indication 'follow up of medication'.

Study indication	Test	Code (LOINC)
Anemia	GGT	2324-2
Anemia	ALT (GPT)	1742-6
Anemia	GFR MDRD	33914-3
Anemia	GFR CKD-EPI	62238-1
Anemia	Creatinine	2160-0
Anemia	TSH	3016-3
Anemia	TSH	11580-8
Anemia	RBC	789-8
Anemia	MCH	785-6
Anemia	MCHC	786-4
Anemia	MCV	787-2
Anemia	Hct	789-8
Anemia	Hct	11273-0
Anemia	Hct automated	4544-3
Anemia	Hb	718-7
Anemia	Hb	785-6
Anemia	RBC distribution	788-0
Anemia	WBC	6690-2
Anemia	WBC formula	49024-3
Anemia	WBC morphology	18314-5
Anemia	WBC leucocytes #	6690-2
Anemia	WBC monocytes %	5905-5
Anemia	WBC monocytes/leuco	26485-3
Anemia	WBC monocytes #	26484-6
Anemia	WBC monocytes # automatc	742-7
Anemia	WBC lymphocytes %	26478-8
Anemia	WBC lymphocytes/leuco	26578-8
Anemia	WBC lymphocytes/leuco autmatic	736-9
Anemia	WBC lymphocytes #	26474-7
Anemia	WBC lymphocytes # autmatc	731-0

Anemia	WBC basophiles #	704-7
Anemia	WBC basophiles %	26444-0
Anemia	WBC basophiles/leuco	30180-4
Anemia	WBC basophiles/100 leuco	706-2
Anemia	WBC eosinophiles #	711-2
Anemia	WBC eosinophiles %	26449-9
Anemia	WBC eosinophiles/leuco	26450-7
Anemia	WBC eosinophiles/100 leuco	713-8
Anemia	WBC neutrophiles #	751-8
Anemia	WBC segm neutrophiles #	30451-9
Anemia	WBC segm neutrophiles/leuco	26505-8
Anemia	WBC segm neutrophiles/leuco autmatic	32200-8
Anemia	WBC normoblasts/leuco	33990-3
Anemia	WBC normoblasts #	715-3
Anemia	WBC meylocytes #	30446-9
Anemia	WBC promyelocytes	26523-1
Anemia	WBC promyelocytes/leuco	26524-9
Anemia	WBC metamyelocyten #	30433-7
Anemia	WBC metamyelocyten/leuco	28541-1
Anemia	Thrombocytes	777-3
Anemia	Leuco + Thrombo morphology	48705-8
Anemia	Ferritin	2276-4
Anemia	CRP	1988-5
Anemia	TIBC	2500-7
Anemia	Iron saturation	2502-3
Anemia	Transferrin	3034-6
Anemia	Transferrin %	48495-6
Anemia	Serum Fe	2498-4
Anemia	Reticulocytes automatic	17849-1
Anemia	Reticulocytes automatic #	60474-4
Anemia	Reticulocytes	4679-7
Anemia	LDH	14804-9
Anemia	Vit B12	2132-9
Anemia	Folate in serum	2284-8
Anemia	Folate in RBC	2283-0

Table 8: Appropriate test for the indication 'anemia'.

Study indication	Test	Code (LOINC)

Liver disease	Cholesterol total	2093-3
Liver disease	Cholesterol HDL	2085-9
Liver disease	Cholesterol total/HDL ratio	32309-7
Liver disease	Cholesterol total/HDL ratio	9830-1
Liver disease	Triglycerides	2571-8
Liver disease	Cholesterol non-HDL	43396-1
Liver disease	Cholesterol LDL calc	13457-7
Liver disease	Glucose post meal	16915-1
Liver disease	Glucose 2h post meal	6689-4
Liver disease	Glucose fasting	1558-6
Liver disease	Glucose serum	2345-7
Liver disease	GGT	2324-2
Liver disease	ALT (GPT)	1742-6
Liver disease	Ferritin	2276-4
Liver disease	AST (GOT)	1920-8
Liver disease	TIBC	2500-7
Liver disease	Iron saturation	2502-3
Liver disease	Transferrin	3034-6
Liver disease	Transferrin %	48495-6
Liver disease	Serum Fe	2498-4
Liver disease	AP	6768-6
Liver disease	Bilirubin conjugated	15152-2
Liver disease	Bilirubin unconjugated	1971-1
Liver disease	Bilirubin direct	1968-7
Liver disease	Bilirubin indirect	1971-1
Liver disease	Bilirubin total	1975-2
Liver disease	Amylase	1798-8
Liver disease	HBsAg IA	5193-8
Liver disease	HBsAG serum	5195-3
Liver disease	HBsAg	16935-9
Liver disease	HAV IgM	13952-7
Liver disease	HAV IgM serum	22314-9
Liver disease	HAV IgM IA	5181-3
Liver disease	HAV IgG IA	40724-7
Liver disease	HAV IgG	22313-1
Liver disease	HAV IgG and IgM	78444-7
Liver disease	Anti-HCV	13955-0
Liver disease	HCV Ab signal/cutoff	48159-8

Liver disease	HCV Ab serum	5198-7
Liver disease	INR Coag assay	34714-6
Liver disease	INR Coag assay in serum	6301-6
Liver disease	PT	5894-1
Liver disease	Albumin	1751-7
Liver disease	EBV IgM	20491-7
Liver disease	EBV IgM IA	24115-8
Liver disease	EBV IgG IA	30083-0
Liver disease	EBV IgG nuclear	31374-2
Liver disease	EBV IgG nuclear IA	5156-5
Liver disease	EBV IgG	24114-1
Liver disease	EBV IgM + IgG	72206-6
Liver disease	CMV IgG	13949-3
Liver disease	CMV IgM IA	24119-0
Liver disease	CMV IgM + IgG	22249-7
Liver disease	CMV IgG IA	5124-3
Liver disease	CMV IgM Ab IA	5126-8
Liver disease	Transferrin %	48495-6

Table 9: Appropriate tests for the indication 'liver disease'.

Study indication	Test	Code (LOINC)
Gout	GFR MDRD	33914-3
Gout	GFR CKD-EPI	62238-1
Gout	Creatinine	2160-0
Gout	Urate crystals	5816-4
Gout	Uric acid (Urate)	3084-1
Gout	Cholesterol non-HDL	43396-1

Table 10: Appropriate tests for the indication 'gout'.

Study indication	Test	Code (LOINC)
Chronic kidney disease	Cholesterol total	2093-3
Chronic kidney disease	Cholesterol HDL	2085-9
Chronic kidney disease	Cholesterol total/HDL ratio	32309-7
Chronic kidney disease	Cholesterol total/HDL ratio	9830-1
Chronic kidney disease	Triglycerides	2571-8
Chronic kidney disease	Cholesterol non-HDL	43396-1
Chronic kidney disease	Cholesterol LDL calc	13457-7
Chronic kidney disease	Glucose post meal	16915-1

Chronic kidney disease	Glucose 2h post meal	6689-4
Chronic kidney disease	Glucose fasting	1558-6
Chronic kidney disease	Glucose serum	2345-7
Chronic kidney disease	GFR MDRD	33914-3
Chronic kidney disease	GFR CKD-EPI	62238-1
Chronic kidney disease	Creatinine	2160-0
Chronic kidney disease	Protein/Creatinine ratio (urine)	16285-9
Chronic kidney disease	Microalbumin (urine)	14957-5
Chronic kidney disease	Albumine/Creatinine Ratio (urine)	14959-1
Chronic kidney disease	RBC	789-8
Chronic kidney disease	MCH	785-6
Chronic kidney disease	MCHC	786-4
Chronic kidney disease	MCV	787-2
Chronic kidney disease	Hct	789-8
Chronic kidney disease	Hct	11273-0
Chronic kidney disease	Hct automated	4544-3
Chronic kidney disease	Hb	718-7
Chronic kidney disease	Hb	785-6
Chronic kidney disease	RBC distribution	788-0
Chronic kidney disease	PTH	2731-8
Chronic kidney disease	Calcium (Ca)	2000-8
Chronic kidney disease	25-OH-Vitamin D	1990-1
Chronic kidney disease	25-OH-Vitamin D2 + D3	83070-3
Chronic kidney disease	Phosphate	14879-1
Chronic kidney disease	Bicarbonate	1963-8

Table 11: Appropriate tests for the indication 'chronic kidney disease'.

**Study indication**	**Test**	**Code (LOINC)**
Acute coronary syndrome	Troponin	10839-9
Acute coronary syndrome	Troponin T	6598-7
Acute coronary syndrome	Troponin I	49563-0
Lung embolism	D-dimer	48065-7

Table 12: Appropriate tests for the indications 'acute coronary syndrome' and 'lung embolism'.

**Study indication**	**Test**	**Code (LOINC)**
Rheumatoid arthritis	ALT (GPT)	1742-6
Rheumatoid arthritis	GFR MDRD	33914-3
Rheumatoid arthritis	GFR CKD-EPI	62238-1

Rheumatoid arthritis	Creatinine	2160-0
Rheumatoid arthritis	RBC	789-8
Rheumatoid arthritis	MCH	785-6
Rheumatoid arthritis	MCHC	786-4
Rheumatoid arthritis	MCV	787-2
Rheumatoid arthritis	Hct	789-8
Rheumatoid arthritis	Hct	11273-0
Rheumatoid arthritis	Hct automated	4544-3
Rheumatoid arthritis	Hb	718-7
Rheumatoid arthritis	Hb	785-6
Rheumatoid arthritis	RBC distribution	788-0
Rheumatoid arthritis	WBC	6690-2
Rheumatoid arthritis	WBC formula	49024-3
Rheumatoid arthritis	WBC morphology	18314-5
Rheumatoid arthritis	WBC leucocytes #	6690-2
Rheumatoid arthritis	WBC monocytes %	5905-5
Rheumatoid arthritis	WBC monocytes/leuco	26485-3
Rheumatoid arthritis	WBC monocytes #	26484-6
Rheumatoid arthritis	WBC monocytes # automatc	742-7
Rheumatoid arthritis	WBC lymphocytes %	26478-8
Rheumatoid arthritis	WBC lymphocytes/leuco	26578-8
Rheumatoid arthritis	WBC lymphocytes/leuco autmatic	736-9
Rheumatoid arthritis	WBC lymphocytes #	26474-7
Rheumatoid arthritis	WBC lymphocytes # autmatc	731-0
Rheumatoid arthritis	WBC basophiles #	704-7
Rheumatoid arthritis	WBC basophiles %	26444-0
Rheumatoid arthritis	WBC basophiles/leuco	30180-4
Rheumatoid arthritis	WBC basophiles/100 leuco	706-2
Rheumatoid arthritis	WBC eosinophiles #	711-2
Rheumatoid arthritis	WBC eosinophiles %	26449-9
Rheumatoid arthritis	WBC eosinophiles/leuco	26450-7
Rheumatoid arthritis	WBC eosinophiles/100 leuco	713-8
Rheumatoid arthritis	WBC neutrophiles #	751-8
Rheumatoid arthritis	WBC segm neutrophiles #	30451-9
Rheumatoid arthritis	WBC segm neutrophiles/leuco	26505-8
Rheumatoid arthritis	WBC segm neutrophiles/leuco autmatic	32200-8
Rheumatoid arthritis	WBC normoblasts/leuco	33990-3
Rheumatoid arthritis	WBC normoblasts #	715-3

Rheumatoid arthritis	WBC meylocytes #	30446-9
Rheumatoid arthritis	WBC promyelocytes	26523-1
Rheumatoid arthritis	WBC promyelocytes/leuco	26524-9
Rheumatoid arthritis	WBC metamyelocyten #	30433-7
Rheumatoid arthritis	WBC metamyelocyten/leuco	28541-1
Rheumatoid arthritis	Thrombocytes	777-3
Rheumatoid arthritis	Leuco + Thrombo morphology	48705-8
Rheumatoid arthritis	CRP	1988-5
Rheumatoid arthritis	Anti-CCP IgG	33935-8
Rheumatoid arthritis	Anti-CCP	32218-0
Rheumatoid arthritis	RF IA	6928-6
Rheumatoid arthritis	RF	11572-5

Table 13: Appropriate tests for the indication 'rheumatoid arthritis'.

Study indication	Test	Code (LOINC)
Thyroid disease	TSH	3016-3
Thyroid disease	TSH	11580-8
Thyroid disease	WBC formula	49024-3
Thyroid disease	WBC morphology	18314-5
Thyroid disease	WBC leucocytes #	6690-2
Thyroid disease	WBC monocytes %	5905-5
Thyroid disease	WBC monocytes/leuco	26485-3
Thyroid disease	WBC monocytes #	26484-6
Thyroid disease	WBC monocytes # automatc	742-7
Thyroid disease	WBC lymphocytes %	26478-8
Thyroid disease	WBC lymphocytes/leuco	26578-8
Thyroid disease	WBC lymphocytes/leuco autmatic	736-9
Thyroid disease	WBC lymphocytes #	26474-7
Thyroid disease	WBC lymphocytes # autmatc	731-0
Thyroid disease	WBC basophiles #	704-7
Thyroid disease	WBC basophiles %	26444-0
Thyroid disease	WBC basophiles/leuco	30180-4
Thyroid disease	WBC basophiles/100 leuco	706-2
Thyroid disease	WBC eosinophiles #	711-2
Thyroid disease	WBC eosinophiles %	26449-9
Thyroid disease	WBC eosinophiles/leuco	26450-7
Thyroid disease	WBC eosinophiles/100 leuco	713-8
Thyroid disease	WBC neutrophiles #	751-8

Thyroid disease	WBC segm neutrophiles #	30451-9
Thyroid disease	WBC segm neutrophiles/leuco	26505-8
Thyroid disease	WBC segm neutrophiles/leuco autmatic	32200-8
Thyroid disease	WBC normoblasts/leuco	33990-3
Thyroid disease	WBC normoblasts #	715-3
Thyroid disease	WBC meylocytes #	30446-9
Thyroid disease	WBC promyelocytes	26523-1
Thyroid disease	WBC promyelocytes/leuco	26524-9
Thyroid disease	WBC metamyelocyten #	30433-7
Thyroid disease	WBC metamyelocyten/leuco	28541-1
Thyroid disease	FT4	14920-3
Thyroid disease	CRP	1988-5
Thyroid disease	FT3	14928-6
Thyroid disease	Anti-TPO-Ab	18332-7
Thyroid disease	TSH-R-Ab (TSI)	30567-2
Thyroid disease	TSH-R-Ab (TSI) serum	5385-0

Table 14: Appropriate tests for the indication 'thyroid disease'.

Study indication	Test	Code (LOINC)
Sexually transmitted infections	HBsAg IA	5193-8
Sexually transmitted infections	HBsAG serum	5195-3
Sexually transmitted infections	HBsAg	16935-9
Sexually transmitted infections	Chlamydia PCR urine	6357-8
Sexually transmitted infections	Chlamydia PCR	21613-5
Sexually transmitted infections	Gonorrhea PCR	21416-3
Sexually transmitted infections	Gonorrhea unspec	24111-7
Sexually transmitted infections	Treponema serum	22587-0
Sexually transmitted infections	Treponema hemaglut	26009-1
Sexually transmitted infections	TP Ig IA	63464-2
Sexually transmitted infections	TP agluttinine	71793-4
Sexually transmitted infections	RPR	31147-2
Sexually transmitted infections	VDRL	50690-7
Sexually transmitted infections	HIV 1 & 2 antigen and antibody (combo)	56888-1
Sexually transmitted infections	HIV 1 & 2 antigen IA	58900-2
Sexually transmitted infections	HIV 1 p24 Ag	9821-0
Sexually transmitted infections	Trichomonas Ag	31978-0
Sexually transmitted infections	HSV culture	5856-0

Table 15: Appropriate tests for the indication 'sexually transmitted infections'.

Study indication	Test	Code (LOINC)
Acute diarrhea	GFR MDRD	33914-3
Acute diarrhea	GFR CKD-EPI	62238-1
Acute diarrhea	Creatinine	2160-0
Acute diarrhea	Sodium	2951-2
Acute diarrhea	RBC	789-8
Acute diarrhea	MCH	785-6
Acute diarrhea	MCHC	786-4
Acute diarrhea	MCV	787-2
Acute diarrhea	Hct	789-8
Acute diarrhea	Hct	11273-0
Acute diarrhea	Hct automated	4544-3
Acute diarrhea	Hb	718-7
Acute diarrhea	Hb	785-6
Acute diarrhea	RBC distribution	788-0
Acute diarrhea	Fecal culture	634-6
Acute diarrhea	Fecal culture	625-4
Acute diarrhea	EHEC	21262-1
Acute diarrhea	Giardia lamblia	31830-3
Acute diarrhea	Microscopy parasites	10701-1
Acute diarrhea	Rotavirus Ab	72174-6
Acute diarrhea	Adenovrius Ab	78529-5
Acute diarrhea	Clostridium difficile	34713-8
Acute diarrhea	Urea	3091-6

Table 16: Appropriate tests for the indication 'acute diarrhea'.

Study indication	Test	Code (LOINC)
Chronic diarrhea	RBC	789-8
Chronic diarrhea	MCH	785-6
Chronic diarrhea	MCHC	786-4
Chronic diarrhea	MCV	787-2
Chronic diarrhea	Hct	789-8
Chronic diarrhea	Hct	11273-0
Chronic diarrhea	Hct automated	4544-3
Chronic diarrhea	Hb	718-7
Chronic diarrhea	Hb	785-6
Chronic diarrhea	RBC distribution	788-0
Chronic diarrhea	WBC	6690-2

Chronic diarrhea	CRP	1988-5
Chronic diarrhea	Fecal culture	634-6
Chronic diarrhea	Giardia lamblia	31830-3
Chronic diarrhea	Clostridium difficile	34713-8
Chronic diarrhea	Cryptosporidium	20781-1
Chronic diarrhea	Calprotectine	38445-3
Chronic diarrhea	IgA total	2458-8
Chronic diarrhea	IgA tTGA	46128-5

Table 17: Appropriate tests for the indication 'chronic diarrhea'.

10.1.2. Secondary outcome diagnostic error

This outcome is defined in 2 independent ways.

The first is based on the physician's evaluation. During data collection, for every new diagnosis, physicians were asked whether it was related to the laboratory panel and, if so, whether the diagnosis was made correctly and timely. If the physician gives a negative answer to this question, this diagnosis is marked as 'possible diagnostic error'.

The second definition is based on a theoretical evaluation, by two independent researchers, of all occurring combinations of new diagnoses and indications for lab panels. For each combination an algorithm (see figure 2) will be applied in order to become an evaluation of 'no' or 'possible' diagnostic error. In case of dispute, a third independent researcher will be asked to resolve the evaluation. The researchers involved in this theoretical evaluation will be blinded to the allocation of the intervention until all evaluations have been made and validated.

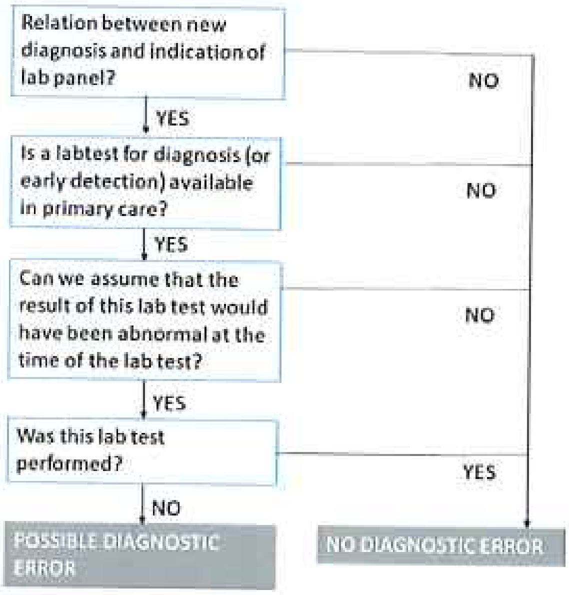

Figure 3: Algorithm used for the theoretical evaluation of diagnostic error.

10.2. Analysis methods

10.2.1. Primary outcome analysis

Appropriateness

For the definition of the primary outcome, three numbers are relevant: (a) the number of requested tests which are appropriate, (b) the number of requested tests which are inappropriate and (c) the number of inappropriately not-requested tests. As indicated in section 5b, the latter number is only relevant for diabetes mellitus, chronic kidney disease, rheumatoid arthritis and thyroid disease. Per patient, aggregated over panels if multiple panels are available, the primary outcome is defined by the the ratio (a)/(a+b+c). We will refer to this ratio as the proportion of appropriate tests in the remainder.

To assess differences between the allocated groups in the proportion appropriate tests, a logistic generalized estimating equation (GEE) model will be used: of interest are the marginal proportions, not the proportions on patient, physician or PCP level.

The logistic GEE model will include the allocated group and laboratory as factors and PCP as the clustering variable. The effect of the intervention will be expressed as the difference in proportions and will be presented together with its associated 95% confidence interval. The proportion of appropriate tests in the two allocated groups will also be estimated from the GEE model and presented with their 95% confidence intervals.

Appropriateness for the composite of all study tests will be compared between intervention and control groups. Furthermore, an additional analysis will be performed that only includes patients who have no indications in addition to the 17 study indications. This additional analysis will correct for an overestimation of inappropriate tests when more than one indication is selected, including indications not under evaluation. These tests would be considered inappropriate even though they could be appropriate according to one of the other indications not being evaluated.

The analyses will be performed on all patients from all physicians according to their allocated group.

Appropriateness for each study indication separately will be performed as a secondary analysis. In this analysis we will include the number of additional indications for the panel as a factor in the analysis.

10.2.2. Secondary outcome analysis

Diagnostic error

The proportion of patients with a missed diagnosis will be analysed by means of a logistic GEE model that includes allocated group and laboratory as factors and uses PCP as the clustering variable. An independent working correlation matrix will be used. The proportion of patients with a missed diagnosis and associated 95% confidence intervals will be estimated from the model.

The difference in proportions will be obtained by subtracting the two proportions. The associated standard error will be calculated from the rules for the variance of a difference between two independent estimates. The 95% confidence interval for the difference will be calculated.

The non-inferiority limit for missed diagnoses is 1%, i.e. the intervention will be deemed non-inferior if the difference between the allocated groups (intervention – control) is less than 1%. Therefore, the intervention will be deemed non-inferior if the upper limit of the 95% confidence interval lies below 1.

As for the primary endpoint, the analysis will be performed for all 17 study indications together. An additional analysis will be performed that only includes patients who have no indications in addition to the 17 study indications

Test volume

The total number of tests will be analysed using a GEE model for count data (Poisson or Negative Binomial to handle potential overdispersion) that includes allocated group and laboratory as factors in the model and PCP as clustering variable. No offset will be used. The number of tests per patient for each group will be estimated from the model and presented together with their associated 95%

confidence intervals. The effect of the intervention will be presented as the ratio between the two numbers with its 95% confidence interval. Statistical significance will be assessed at a significance level of 5%.

10.2.3. Exploratory outcome analysis

The total number of cascades per patient will be analysed using the same methodology as for the total number of tests (test volume, see supra).

10.3. Missing data

For about 15% of patients included in the study, no eForm is available (preliminary figures). This measure of missing data is due to technical difficulties in the pseudonymized data collection. No methods for data imputation are foreseen. Patients for whom no data was collected will be excluded from the data analysis. The exact measure of missing data will be reported in the final report.

10.4. Statistical software

Statistical analyses will be performed with SAS Enterprise Guide software (Copyright © 2019 SAS Institute Inc.). SAS and all other SAS Institute Inc. product or service names are registered trademarks or trademarks of SAS Institute Inc., Cary, NC, USA.

11. Data archiving

12. References

1. Delvaux N, Aertgeerts B, De Sutter A, Ramaekers D, Fieuws S, Belmans A, et al. The effect of evidence-based order sets within a CPOE (computerised physician order entry) system on the quantity and quality of laboratory test ordering in family practice: a cluster randomised trial. Leuven, Belgium: UZ Leuven; 2017.

2. Delvaux N, De Sutter A, Van de Velde S, Ramaekers D, Fieuws S, Aertgeerts B. Electronic Laboratory Medicine ordering with evidence-based Order sets in primary care (ELMO study): protocol for a cluster randomised trial. Implement Sci IS. 2017;12:147.

3. Gamble C, Krishan A, Stocken D, Lewis S, Juszczak E, Doré C, et al. Guidelines for the Content of Statistical Analysis Plans in Clinical Trials. JAMA. 2017;318:2337–43.

4. Delvaux N, Aertgeerts B, van Bussel JC, Goderis G, Vaes B, Vermandere M. Health Data for Research Through a Nationwide Privacy-Proof System in Belgium: Design and Implementation. JMIR Med Inform. 2018;6:e11428.

5. Corilus MedDBase codes. https://www.corilus.be/general-practitioner/meddbase.

6. Forrey A, Mcdonald C, Demoor G, Huff S, Leavelle D, Leland D, et al. Logical observation identifier names and codes (LOINC) database: A public use set of codes and names for electronic reporting of clinical laboratory test results. Clin Chem. 1996;42:81–90.

7. Reference Table Manager (ReTaM).

8. Bentsen BG. International classification of primary care. Scand J Prim Health Care. 1986;4:43–50.

9. Zhi M, Ding EL, Theisen-Toupal J, Whelan J, Arnaout R. The Landscape of Inappropriate Laboratory Testing: A 15-Year Meta-Analysis. PLoS ONE. 2013;8:e78962.

10. Health and Social Care Directorate Quality standards Process guide. Manchester, UK: National Institute for Health and Care Excellence (NICE); 2016. https://www.nice.org.uk/Media/Default/Standards-and-indicators/Quality-standards/quality-standards-process-guide.pdf.
