# Supplementary material for: Clinical decision support improves the appropriateness of laboratory test ordering in primary care without increasing diagnostic error: the ELMO cluster randomized trial
Source: Implement Sci. 2020 Nov 4;15:100. doi: 10.1186/s13012-020-01059-y (PMC7640389; doi:10.1186/s13012-020-01059-y)
Supplement: Supplementary file 2 — Additional file 2. [file 13012_2020_1059_MOESM2_ESM.docx]

Supplement 2

Results

|  |  | **CDSS arm** | **Control arm** | **Total** |
| --- | --- | --- | --- | --- |
| Number of GPs | | 135 (49.63%) | 137 (50.37%) | 272 |
|  | Age (years, SD) | 41 (13.59) | 41 (13.27) |  |
|  | Experience (years, SD) | 14 (18.81) | 15 (19.40) |  |
|  | Female (%) | 65.00% | 62.14% |  |
| Number of practices | | 36 (50%) | 36 (50.00%) | 72 |
|  | Laboratory 1 | 19 (52.78%) | 20 (55.56%) | 39 |
|  | Laboratory 2 | 5 (13.89%) | 3 (8.33%) | 8 |
|  | Laboratory 3 | 12 (33.33%) | 13 (36.31%) | 25 |

eTable 1: Baseline characteristics of participating GPs


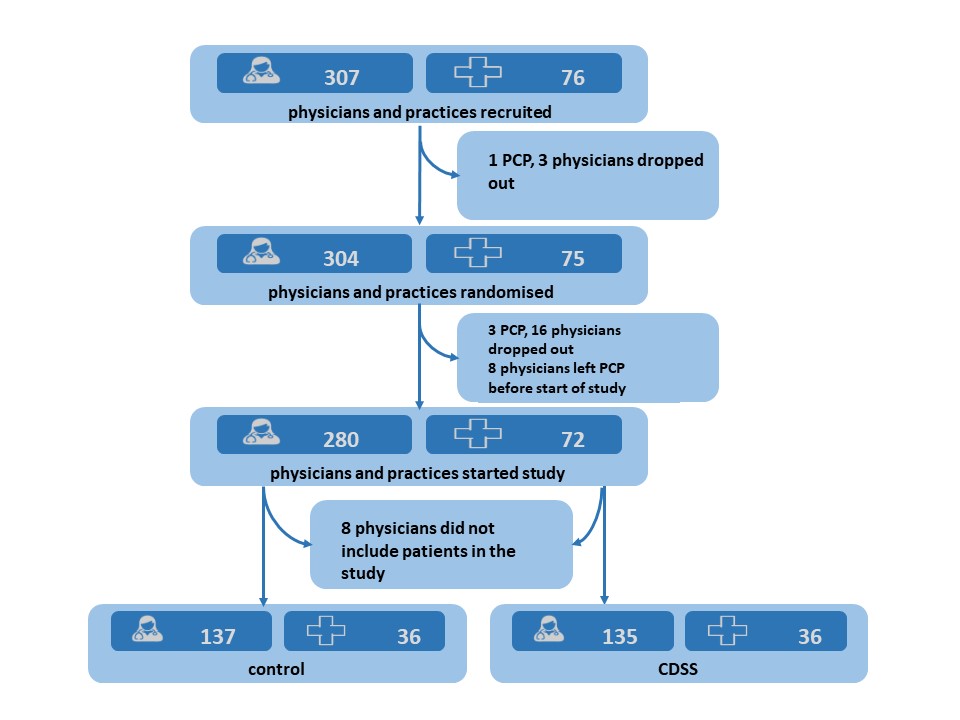


eFigure 1: Flow of GP and practice recruitment.


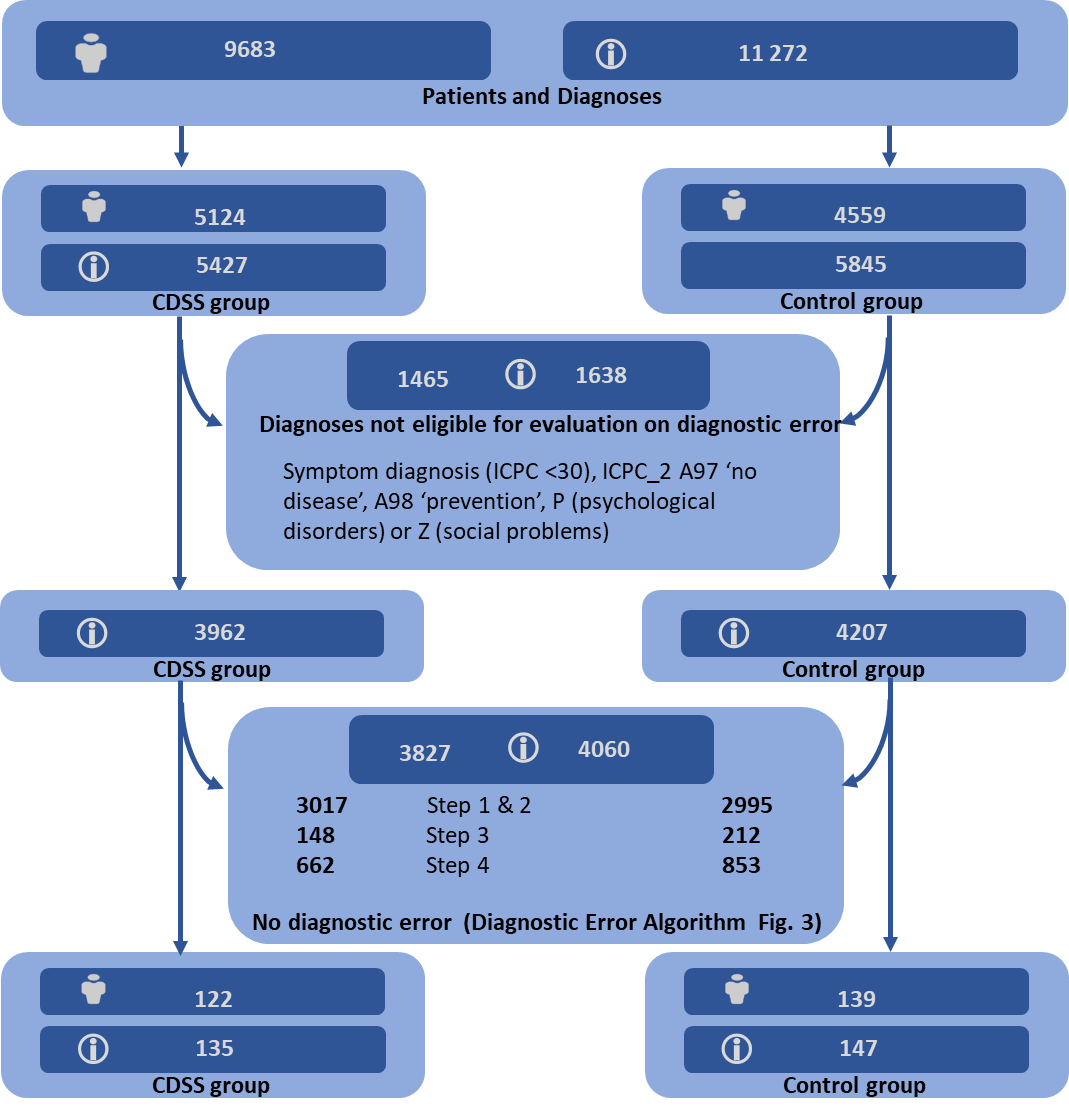


eFigure 2: Flow of patients and new diagnoses for the assessment of possible diagnostic error. CDSS: clinical decision support system; ICPC: International Classification of Primary Care.

Sensitivity analyses

Additional analyses were performed to investigate the effect of the age difference across both arms on the difference in proportions for the primary outcome. The difference in proportions remained robust across all subgroups, increasing slightly in older patients and when omitting outlier practices, and decreasing slightly in younger patients. eTable 2 shows the exact differences in proportions for each subgroup.

|  |  | **CDSS arm** | **Control arm** | | **Difference in proportions** | | **p-value** |
| --- | --- | --- | --- | --- | --- | --- | --- |
| Primary outcome (all tests) – 45 to 65 years | | 0.57 (0.53-0.61) | 0.34 (0.31-0.37) | | 0.23 (0.18-0.29) | | <.0001 |
| Primary outcome (all tests) – ≥ 65 years | | 0.57 (0.53-0.61) | 0.33 (0.30-0.37) | | 0.24 (0.18-0.29) | | <.0001 |
| Primary outcome (all tests) – < 45 years | | 0.64 (0.60-0.68) | 0.49 (0.46-0.53) | | 0.15 (0.10-0.20) | | <.0001 |
| Primary outcome (all tests) – without outliers | | 0.62 (0.59-0.66) | 0.39 (0.35-0.42) | | 0.24 (0.19-0.28) | | <.0001 |
| Primary outcome (population without check-up) | | 0.64 (0.61-0.68) | | 0.52 (0.49-0.54) | | 0.13 (0.08-0.17) | <.0001 |
| Primary outcome (all tests) – with age as a factor in the analysis | | 0.59 (0.55-0.62) | | 0.37 (0.34-0.40) | | 0.21 (0.17-0.26) | <.0001 |

eTable 2: Additional sensitivity analyses by age categories, by omitting outlier primary care practices, and by including age as an additional factor in the prespecified analysis.

We judged that possible documentation bias would have been most pronounced in the group of patients identified as ‘check-up’. For the GPs in the CDSS arm it was very clear which tests were included in this order set, but for GPs in the control arm it was much less clear. Hence, it seems plausible that some patients in the control arm were in fact patients with additional conditions rather than healthy, asymptomatic patients. We analysed that data of all patients that did not have any tests ordered for ‘check-up’ and observed that the intervention effect lessened to 0.11 (95% CI 0.06-0.17), but still remained significant.


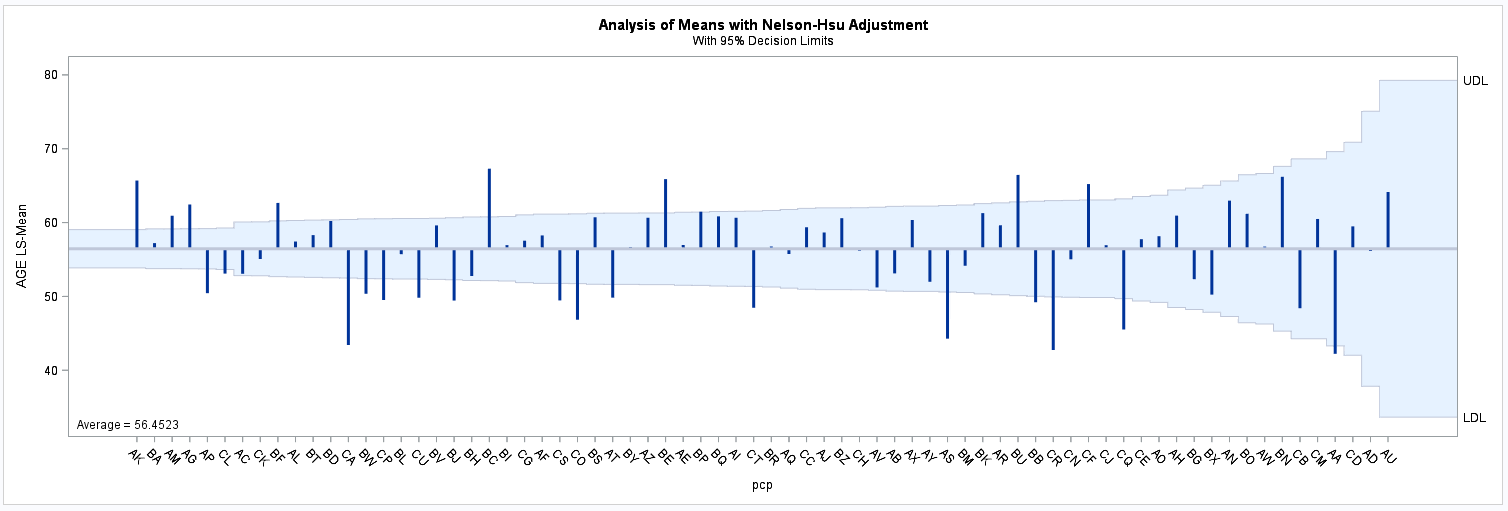


eFigure 3: Funnel plot of mean age of patients per practice (PCP). The practices are arranged per size, where the largest pracitces are on the left side of the X-axis and the smallest practices on the right side. Outlier practices AK and AG were allocated to the CDSS arm. PCP: primary care practice; CDSS: clinical decision support system.


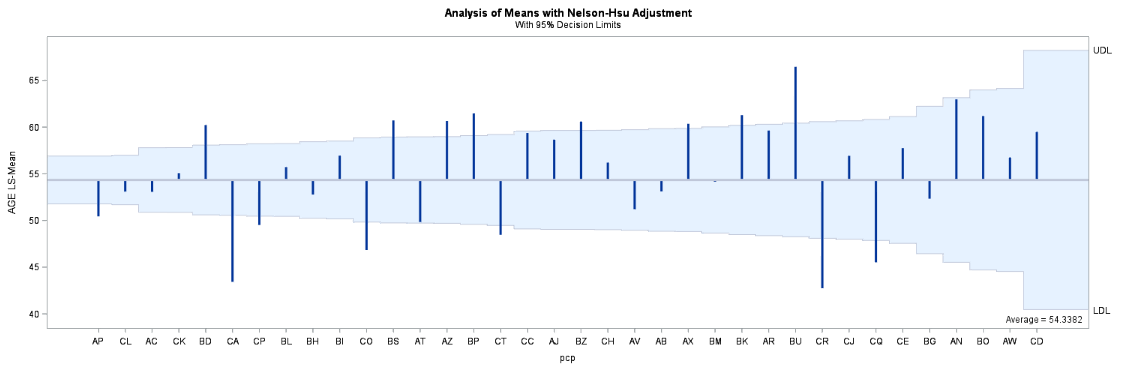


eFigure 4: Funnel plot of control practices only. Practices CA and AP decreased the mean age of patients in the control arm. PCP: primary care practice.


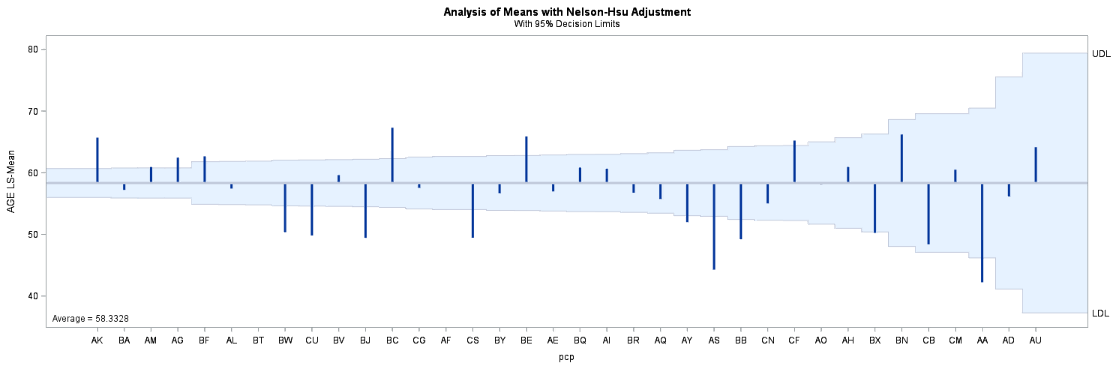


eFigure 5: Funnel plot of intervention practices only. Practices AK and AG increased the mean age of patients in the intervention arm. PCP: primary care practice.

For a series of subgroups, we compared the results of several signal tests to assess the comparability of each group. All the patients in the subgroup were identified by their GP as having the condition, disease of indication. Within these subgroups, the results of tests which were considered pertinent to the evaluation of this condition were compared. For example, in the subgroup of patients with a thyroid disease, the mean TSH values were compared between control and CDSS arm. eTable 3 summarizes the results of the signal tests for each of the subgroups. In the subgroup of patients with general check-up as indication, a small difference in glycated hemoglobin (HbA1c) was witnessed. No important differences in thyroid stimulating hormone (TSH) and creatinine values was observed. In the subgroup of patients with type 2 diabetes, no important differences in HbA1c levels were observed. In the subgroup of patients with cardiovascular disease, a small difference in total cholesterol levels was observed. No differences were observed in HbA1c levels. In the subgroup of patients with thyroid disease, a small difference in TSH levels was observed, however both values were within normal ranges. In the subgroup of patients with chronic kidney disease (CKD), a small difference in creatinine levels was observed, indicating that this subgroup of patients in the control arm may have had more pronounced CKD than the patients in the CDSS arm.

| **Indication general check-up** | | | |
| --- | --- | --- | --- |
| **Intervention (1709 patients, 1722 panels)** | | **Control (1922 patients, 1936 panels)** | |
| TSH (n=1215) | | TSH (n=1655) | |
| Mean 1.69 | SD 0.03 | Mean 1.74 | SD 0.05 |
| HbA1c (n=229) | | HbA1c (n=285) | |
| Mean 47.64 | SD 0.95 | Mean 43.57 | SD 0.67 |
| Creatinine (n=1510) | | Creatinine (n=1877) | |
| Mean 0.87 | SD 0.006 | Mean 0.87 | SD 0.004 |
|  |  |  |  |
| **Indication type 2 diabetes** | | | |
| **Intervention (1965 patients, 2160 panels)** | | **Control (901 patients, 953 panels)** | |
| HbA1c (n=1516) | | HbA1c (n=794) | |
| Mean 51.27 | SD 0.32 | Mean 52.30 | SD 0.48 |
|  |  |  |  |
| **Indication cardiovascular disease management** | | | |
| **Intervention (1356 patients, 1381 panels)** | | **Control (558 patients, 585 panels)** | |
| Cholesterol (n=1350) | | Cholesterol (n=515) | |
| Mean 191.51 | SD 1.18 | Mean 197.10 | SD 2.01 |
| HbA1c (n=346) |  | HbA1c (n=117) |  |
| Mean 49.28 | SD 0.71 | Mean 48.57 | SD 0.71 |
|  |  |  |  |
| **Indication thyroid disease** | | | |
| **Intervention (1136 patients, 1164 panels)** | | **Control (550 patients, 576 panels)** | |
| TSH (n=1156) | | TSH (n=576) | |
| Mean 2.01 | SD 0.11 | Mean 2.70 | SD 0.25 |
|  | | | |
| **Indication CKD** | | | |
| **Intervention (574 patients, 587 panels)** | | **Control (141 patients, 168 panels)** | |
| Creatinine (n=579) | | Creatinine (n=154) | |
| Mean 1.06 | SD 0.02 | Mean 1.36 | SD 0.06 |

eTable 3: Subgroup analyses of signal tests for patients and panels including the indications general check-up, type 2 diabetes, cardiovascular disease management, thyroid disease and CKD. TSH: thyroid stimulating hormone; HbA1c: glycated hemoglobin; CKD: chronic kidney disease; SD: standard deviation.
